# LDHA Promotes Oral Squamous Cell Carcinoma Progression Through Facilitating Glycolysis and Epithelial–Mesenchymal Transition

**DOI:** 10.3389/fonc.2019.01446

**Published:** 2019-12-19

**Authors:** Hongshi Cai, Jiaxin Li, Yadong Zhang, Yan Liao, Yue Zhu, Cheng Wang, Jinsong Hou

**Affiliations:** ^1^Department of Oral and Maxillofacial Surgery, Guanghua School of Stomatology, Hospital of Stomatology, Sun Yat-sen University, Guangzhou, China; ^2^Guangdong Provincial Key Laboratory of Stomatology, Sun Yat-sen University, Guangzhou, China

**Keywords:** epithelial–mesenchymal transition, glycolysis, GSEA, LDHA, oral squamous cell carcinoma, oxamate, WGCNA

## Abstract

Aerobic glycolysis is the main pathway for energy metabolism in cancer cells. It provides energy and biosynthetic substances for tumor progression and metastasis by increasing lactate production. Lactate dehydrogenase A (LDHA) promotes glycolysis process by catalyzing the conversion of pyruvate to lactate. Despite LDHA exhibiting carcinogenesis in various cancers, its role in oral squamous cell carcinoma (OSCC) remains unclear. This study demonstrated that LDHA was over-expressed in both OSCC tissues and cell lines, and was significantly associated with lower overall survival rates in patients with OSCC. Using weighted gene correlation network analysis and gene set enrichment analysis for the gene expression data of patients with OSCC (obtained from The Cancer Genome Atlas database), a close association was identified between epithelial–mesenchymal transition (EMT) and LDHA in promoting OSCC progression. The knockdown of LDHA suppressed EMT, cell proliferation, and migration and invasion of OSCC cells *in vitro*. Moreover, the silencing of LDHA inhibited tumor growth *in vivo*. Oxamate, as a competitive LDHA inhibitor, was also suppressed diverse malignant biocharacteristics of OSCC cells. Our findings reveal that LDHA acts as an oncogene to promote malignant progression of OSCC by facilitating glycolysis and EMT, and LDHA may be a potential anticancer therapeutic target.

## Introduction

Oral cancer is the most common location for head and neck cancers. Among all oral cancer subtypes, oral squamous cell carcinoma (OSCC) is the most common (1). Despite the existence of mature diagnostic and comprehensive treatment methods, the 5-year survival rate of patients with OSCC remains relatively poor, and ~50% of patients with OSCC die within 5 years. The main causes of the poor prognosis of OSCC are early local invasion and distant metastasis (2).

Aerobic glycolysis, i.e., the Warburg effect, constitutes one of its important features during the development of OSCC (3–5). Cancer cells enhance glucose uptake and lactate production through aerobic glycolysis to meet energy metabolism and biosynthesis needs. Aerobic glycolysis can increase the lactate content of cancer cells and reduce the pH of the tumor microenvironment. As an important carcinogen, lactate is closely associated with tumor growth, immune escape, angiogenesis and epithelial–mesenchymal transition (EMT) (6–8). In the glycolytic pathway, lactate dehydrogenase A (LDHA) irreversibly catalyzes the conversion of pyruvate to lactate via the oxidative dehydrogenation of nicotinamide adenine dinucleotide (NADH) to NAD+. Several studies have reported that LDHA is highly expressed in various cancers such as pancreatic cancer, gastric, gallbladder, and breast cancers, as well as nasopharyngeal and hepatocellular carcinomas; its expression is positively correlated with the malignant progression of the cancers (9–15). However, the relationship between LDHA expression and the progression of OSCC, as well as the underlying mechanisms that affect the biological behavior of OSCC cells, are still unclear.

Weighted gene correlation network analysis (WGCNA) uses up to 10,000 of the most variable genes (or in some cases all genes) to identify the target gene set, construct a weighted correlation network, and identify modules comprising highly related genes (16). By using WGCNA, it can identify the gene module that is highly synergistic with LDHA expression in order to explore some of the biological processes that have undergone changes in patients with OSCC. The enrichment of gene expression in biological functions and pathways can be analyzed using gene set enrichment analysis (GSEA). This approach ranks genes according to the degree of differential expression of the two sample types; subsequent testing establishes whether the predetermined gene sets are enriched at the top or bottom of the ranking table.

In this study, we aimed to detect the expression of LDHA in OSCC and explore its effect on the malignant progression of OSCC. We first demonstrated that LDHA was up-regulated in OSCC. Next, we combined gene expression data with GSEA and WGCNA to confirm that LDHA was associated with the EMT gene set and identified that EMT progression could be inhibited by LDHA silencing. Furthermore, we found that LDHA knockdown suppressed OSCC cell growth both *in vitro* and *in vivo*. Finally, we used a competitive LDHA inhibitor, oxamate (17), which was found to significantly inhibit glycolysis, proliferation, migration and invasion of OSCC cells. Based on these results, it was revealed that LDHA plays an important role in the progression of OSCC through EMT. Therefore, it is proposed that LDHA may be a potential therapeutic target in OSCC.

## Materials and Methods

### OSCC Sample, Cell Lines, and Culture

Eighty-nine OSCC tissues samples, 18 matched adjacent non-cancerous normal tissues (ANCT), and two normal oral mucosal epithelial tissues were collected at the Hospital of Stomatology, Sun Yat-sen University. The study was approved by the hospital's ethics committee and was conducted in accordance with the Declaration of Helsinki's guidelines, between September 2017 and December 2018. All patients provided informed consent to participate in the study. The fresh tissues were then divided into two parts after tumor resection, one of which was stored in a cryotube at −80°C for RNA and protein extraction, and the other was fixed in formalin and embedded in paraffin for immunohistochemical analysis. All staging was determined according to the 7th American Joint Committee on Cancer Staging System.

The human OSCC cell lines SCC1, SCC25, HSC6, SCC15, HSC3, and 293T were obtained from the American Type Culture Collection. The SCC1, HSC6, HSC3, and 293T cell lines were cultured in Dulbecco's modified Eagle's medium (DMEM, Gibco, USA) containing 10% fetal bovine serum (FBS, Gibco, USA). The SCC15 and SCC25 cell lines were grown in DMEM/Ham”s F12 medium (DMEM/F12, Gibco, USA) supplemented with 10% FBS and 400 ng/mL hydrocortisone (H0888, Sigma-Aldrich). Cells were cultured at 37°C under a humidified incubator with 5% CO_2_. For the TGF-β1 (R&D, USA) treatment groups, cells were starved with serum-free media for 24 h and then treated with TGF-β1 (10 ng/ml) for 48 h.

### Immunohistochemistry

For the immunohistochemistry analysis, paraffin-embedded tissues were cut into 3.5-μm sections. They were dewaxed, dehydrated, subjected to antigenic retrieval with 0.01 mol/L sodium citrate buffer (comprising 3 g sodium citrate dihydrate, 0.4 g citric acid monohydrate, and 1,000 ml distilled water; pH = 6.0), and blocked with goat serum (AR0009, BosterBio, China). Tissue sections were incubated with primary antibodies against LDHA (1:250, C4B5, Cell Signaling Technology, USA), ZO1 (TJP1 tight junction protein 1) (1:400, 66452-1-Ig, Proteintech), E-cadherin (1:400, 24E10, Cell Signaling Technology), N-cadherin (1:1,000, D4R1H, Cell Signaling Technology), Vimentin (1:200, D21H3, Cell Signaling Technology), and Slug (1:100, ab128485, Abcam) overnight at 4°C. After staining with diaminobenzidine (DAB, GK600510, Gene Tech, China) and counterstained with hematoxylin (D006, Nanjing Jiancheng Biotech, China), both the proportion of positive cells (0–100%) and the intensity of the cytoplasmic staining (0: no staining, 1: weak, 2: moderate, and 3: strong) were used to calculate the LDHA histo-score. Each slice's H-score was determined by the staining index (the proportion of positive cells × staining intensity) (H-score: 0–300). Tissues with an H-score >150 were classified as the high-expression group, while whereas those with H-scores ≤ 150 were classified as the low-expression group.

### Western Blot Analysis

Cells were lysed with a RIPA buffer (CW2333S, CWbio, China) and a protease inhibitor cocktail set I (539131, Millipore, USA). The cellular proteins' supernatants were collected by centrifugation at 14,000 g for 20 min at 4°C. Protein concentrations were measured with the BCA protein assay kit (CW0014S, CWbio). Equal amounts of proteins were separated by 10% SDS-PAGE (P0014B, CWbio) and were transferred to PVDF membranes (ISEQ00010, Millipore, USA), which were blocked for 1 h with 5% bovine serum albumin (0332, Amresco) and were incubated overnight at 4°C with related primary antibodies, including LDHA (1:1,000, C4B5, Cell Signaling) matrix metalloprotein 2 (MMP2, 1:1,000, ab37150, Abcam), ZO1 (TJP1 tight junction protein 1; 1:1,000, D7D12, Cell Signaling), E-cadherin (1:1,000, 24E10, Cell Signaling), N-cadherin (1:1,000, D4R1H, Cell Signaling), Vimentin (1:1,000, D21H3, Cell Signaling), Slug (1:1,000, C19G7, Cell Signaling), and β-ACTIN (1:1,000, D6A8, Cell Signaling). After probing with secondary antibodies (GK600510, Gene Tech, China), the membranes were conjugated to horseradish peroxidase (HRP) in room temperature for 1 h, and the bands were exposed by a chemiluminescent HRP substrate (WBKLS0500, Millipore, USA). The quantitative analysis of Western blots was carried out by Image J (https://imagej.nih.gov/ij/download.html).

### RNA Isolation and Quantitative Real-Time PCR (qRT-PCR)

Total RNA was extracted from the cells and tissues using RNAzol® RT (RN190, Molecular Research Center, USA) following the manufacturer's instructions. Then, 1 μg of total RNA was reverse-transcribed to complementary DNA using the PrimeScript RT reagent kit (RR036A, Takara Bio, Japan). The qPCR reactions were performed with a SYBR Green Master Mix (11201ES08, Yeasen, China) and were run at 95°C for 5 min, followed by 40 cycles of 95°C for 10 s, 60°C for 20 s, and 72°C for 20 s, with a final cycle of 95°C for 15 s, 60°C for 60 s, and 95°C for 15 s as the melting curve in the LightCycler 96 System (Roche, Germany). LDHA expression was calculated by 2^−ΔΔCt^ normalized to β-ACTIN. The primer sequences used were as follow: LDHA-forward: 5′-GAGTGGAATGAATGTTGCTGGTGTC-3′; LDHA- reverse: 5′-CCAGGATGTGTAGCCTTTGAGTTTG-3′; TJP1-forward: 5′- CTGCCAGTGTCATTTACATCCTTCT-3′; TJP1-reverse: 5′- GCTGTCCCTCCGCTGATACCT-3′; CDH1-forward: 5′- AGCACCTTCCATGACAGACCC-3′; CDH1-reverse: 5′- AGAACGCATTGCCACATACAC-3′; CDH2-forward: 5′- CATCATCATCCTGCTTATCCTTGT-3′; CDH2-reverse: 5′- GGTCTTCTTCTCCTCCACCTTCTT-3′; VIM-forward: 5′- ATCGTGATGCTGAGAAGTTTCG-3′; VIM-reverse: 5′- TCTGGATTCACTCCCTCTGGTT-3′; SNAI2-forward: 5′-TTTTGCACTGGTATTTCTTTACATC-3′; SNAI2-reverse: 5′-CCCTGGTTGCTTCAAGGACAC-3′; β-ACTIN-forward: 5′-CTACCTCATGAAGATCCTCACCGA-3′; and β-ACTIN-reverse: 5′-TTCTCCTTAATGTCACGCACGATT-3′.

### Lentiviral Short-Hairpin RNA (shRNA) Transfection

The stable knockdown of LDHA expression was achieved using the lentiviral carrying shRNA process. Lentivirus shLDHAs (shLDHA-1: 5′-GGCAAAGACTATAATGTAA-3′, shLDHA-2: 5′-TAAGGGTCTTTACGGAATA-3′) and a non-specific control shRNA were cloned into the pLVshRNA-EGFP (2A) puro vector. The target plasmids and lentivirus packaging plasmids pGag/Pol, pRev, and pVSV-G were co-transfected with 293T cells according the manufacturer's instructions (GenePharma, China). The virus-containing supernatant was collected, filtered, and infected HSC3, SCC15 cells with 5 μg/ml Polybrene (H8761, solarbio, China). The infected cells were screened with puromycin (HSC3: 3 μg/ml, SCC15: 8 μg/ml) (P8230, solarbio, China) for 15 days.

### Lactate Production and Glucose Consumption Measurement

HSC3 and SCC15 cells were seeded in 6-well plates at 2.5 × 10^5^ cells per well and a well without cells as the control. After the cells were attached, they were switched to a complete medium. The medium was collected at 24 h of incubation to determine lactate production and glucose consumption. The respective concentrations of lactate production and glucose consumption were measured with commercial kits (a lactate assay kit [A019] from Nanjing Jiancheng Biotech, and a glucose assay kit [E1010] from Applygen, China) according to the manufacturer's instructions.

### Cell Proliferation and Colony Formation Assay

HSC3 and SCC15 cells were seeded in 96-well plates at a density of 2,000 cells per well. Cell proliferation was detected using a Cell Counting Kit-8 (CCK-8, 40203ES80, Yeasen, China) at 0, 24, 48, 72, and 96 h. In each well, 100 μl of 10% CCK-8 reagent (10 μl CCK-8 and 90 μl serum-free media) was added, and the plates were incubated for 1 h at 37°C. The absorbance values were measured at 450 nm using a microplate reader (Bio-Rad, USA), and the growth curve was plotted based on absorbance and time.

The colony formation assay was conducted by inoculating 10,000 HSC3 cells or 500 SCC15 cells in 6-well plates and fixing and staining after 7–10 days of culture. The number of HSC3 cell colonies were counted under a microscope at 5 × magnification by selecting three random fields. Colony numbers in the SCC15 cells were directly enumerated.

### Wound-Healing, Migration, and Invasion Assays

For the wound-healing assay, HSC3 and SCC15 cells were seeded and cultured up to 90% of the coverage area in 6-well plates. The cells were scraped with a sterile pipette tip, washed twice with phosphate-buffered saline (PBS), and treated with FBS-free media for 24 h. Five fields of view were randomly selected under a microscope with 100 × magnification. The distances between two cell edges were quantified using Image J and were used to calculate the wound healing rate. A Transwell assay of cell migration and invasion was conducted using 5 × 10^4^ HSC3 cells or 1.4 × 10^5^ SCC15 cells in 200 μl FBS-free media. They were plated on the upper chambers of the Transwell (for cell migration assay, 8-μm pore size) (Corning, China), and Transwell coated with 0.33 mg/ml Matrigel (for cell invasion assay, 10 mg/ml, 354234, Corning, China), while 600 μl of complete media was added to the lower chamber. After a 24-h incubation period, cells that passed through the chamber filter and attached to the surface of the filter were fixed, stained with hematoxylin, and counted under a 100× microscope in five random fields per chamber according to the number of nuclei.

### LDH Activity Assay

HSC3 and SCC15 cells were seeded and cultured in 96-well plates to a density of nearly 80%; oxamate was treated for 24 h. The lactate dehydrogenase (LDH) activity of the cells was determined using the LDH Assay Kit (C0017, Beyotime, China), in accordance with the manufacturer's instructions. Absorbance values were detected using a Bio-Rad microplate reader at 490 nm with a reference wavelength of 600 nm.

### Tumor Xenograft

Female NOD/SCID mice (5–6 weeks of age) were randomly distributed into three groups: the shNS group, the shLDHA-1 group, and the shLDHA-2 group (*n* = 6 in each group). Approximately 5 × 10^7^ HSC3 cells per mouse were diluted in 200 μl of PBS and were subcutaneously injected into the inner thigh to establish a subcutaneous transplanted model. The transplanted tumors were measured weekly to observe tumor growth. Tumor volume was calculated using the formula V = length × width × width/2. The mice were euthanized 4 weeks after injection, and the tumor xenografts were harvested, photographed, and fixed for immunohistochemical staining. This animal experiment was approved by Sun Yat-sen University's Animal Experiment Ethics Committee.

### The Cancer Genome Atlas (TCGA) Dataset Collection and Data Processing

The head and neck squamous cell carcinoma dataset (566 cases) from TCGA, including preprocessed level 3 RNAseq and clinical data, was downloaded using the UCSC Xena browser on 15 May 2019 (https://xenabrowser.net) (18). Among these cases, clinical samples from the oral cavity (oral tongue, floor of mouth, hard palate, alveolar ridge, oral cavity, and buccle mucosa) comprised 284 OSCC and 30 matched normal oral mucosal epithelial tissues. The gene expression data and survival results of 284 patients with OSCC were used for the survival analysis. Next, the 284 patients with OSCC were divided into high- and low-expression groups based on the median expression levels of LDHA for the GSEA and WGCNA. All the above processes were conducted using R software (R version 3.5.3) and related R packages.

### WGCNA

First, the top 6000 of the most variant genes and OSCC samples were examined by respectively, applying the goodSamplesGenes function and the sample network method to remove the offending genes and samples (Z.K < −2.5). Second, the WGCNA package was applied to construct a gene co-expression network. A soft threshold power was selected based on approximate scale-free topological criteria and was used to calculate the adjacency. Next, the adjacency matrix was transformed into a topological overlap matrix, from which the corresponding dissimilarity was calculated. Third, a hierarchical clustering gene tree was generated using the topological overlap matrix, and genes with similar expression profiles were combined into the same gene module. Fourth, the module eigengene was the first principal component of a particular module and represented the overall level of gene expression within that module. The module eigengenes were calculated to identify modules that were significantly associated with the LDHA expression. According to the significance of the gene network, gene significance was defined to quantify the correlation between the genes and the LDHA expression. A quantitative measure of module membership was defined as the correlation between the gene expression profile and the module eigengene and the intramodular connectivity was defined as the connectivity between a gene and other genes within a module. Finally, gene ontology (GO) and Kyoto Encyclopedia of Genes and Genomes (KEGG) enrichment analyses were performed for the genes in the target modules.

### GSEA

GSEA was conducted between the LDHA high-expression and low-expression groups. The association of LDHA expression with oncogenetic biological function was performed using the Hallmark gene sets. A *p*-value of <0.05 and a false discovery rate (FDR) of <0.05 for a gene set were considered statistically significant.

### Statistical Analysis

The mean and the standard deviation of data were analyzed using GraphPad prism 7.0. software. The Shapiro–Wilk test was used to test normal distribution. Statistical differences between the two groups were analyzed using a two-tailed unpaired or paired Student's *t*-test; for multiple groups, ANOVA with Tukey's multiple comparisons tests were performed. The association between LDHA expression and clinicopathological features in patients with OSCC was conducted using Fisher's exact test. The correlation between genes was analyzed using Pearson's correlation. A *p*-value of <0.05 was considered statistically significant: ^*^p < 0.05, ^**^p < 0.01, ^***^p < 0.001.

## Results

### LDHA Is Up-Regulated in OSCC and Associated With Poor Prognosis

**Figure 1:**
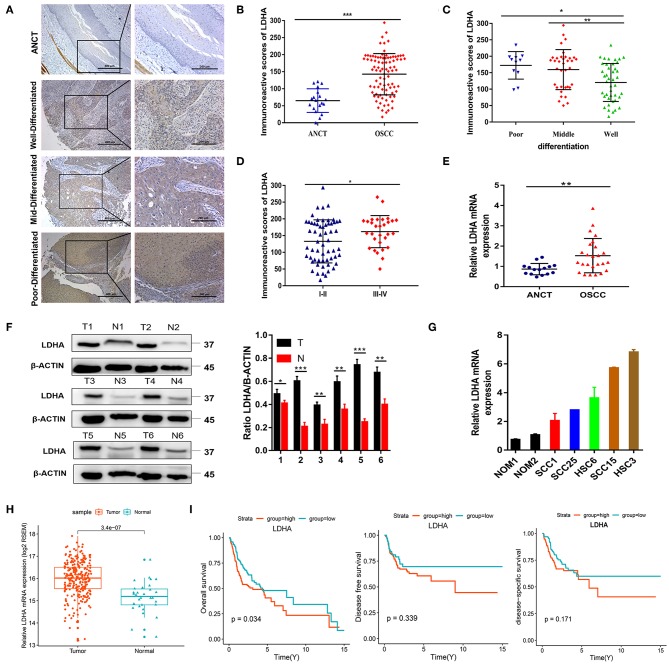
LDHA is up-regulated in OSCC and is associated with poor prognosis. **(A)** Representative immunohistochemical staining images for LDHA in ANCT and pathological differentiation of OSCC tissues. Magnification at 100× (left panel) and 200× (right panel). **(B)** Histological scoring of LDHA in ANCTs and OSCC tissues. **(C)** Histological scoring of LDHA in OSCC tissues with various pathological differentiations. **(D)** Histological scoring of LDHA in OSCC tissues with various TNM classifications. **(E)** Relative LDHA mRNA expression in ANCTs and OSCC tissues. **(F)** LDHA protein expression in six pairs of OSCC tissues and in non-tumor tissues were determined by Western blot analysis (left) and quantitatively analyzed (right). **(G)** Relative expression of LDHA in normal oral mucosal epithelial tissues and OSCC cell lines; **(H)** Relative expression of LDHA mRNA in OSCC tissues (*n* = 284) and matched ANCTs (*n* = 30) from TCGA database. **(I)** Kaplan–Meier survival curves of overall survival, disease-free survival, and disease-specific survival based on patients with OSCC with high- and low-expression LDHA. Differences between the two groups were compared using a log-rank test. The experiment was repeated three times; error bars indicate standard deviation. *p < 0.05, **p < 0.01, ***p < 0.001.

To detect the expression level of LDHA in OSCC, immunohistochemical staining was performed for 89 OSCC tumor tissues and 18 ANCTs. Depending on the Histo-score, it was revealed that OSCC tissue demonstrated a higher staining score for LDHA for ANCT (Figures 1A,B). In addition, LDHA with a poor degree of differentiation compared to a good degree of differentiation middle-differentiated and well-differentiated LDHA was also highly expressed (Figure 1C). Patients with TNM III-IV had similar results compared with patients with TNM I-II (Figure 1D). Further analysis showed that LDHA expression was associated with the degree of differentiation and T stage of the OSCC. However, gender, age, TNM stage, and lymph-node metastasis did not differ between patients with high and low LDHA expression (Table 1). RT-qPCR analysis of LDHA mRNA levels in 25 OSCC tumor tissues and 15 ANCTs revealed that LDHA mRNA levels were elevated in the tumor tissues of patients with OSCC (Figure 1E). Additionally, Western blot analysis indicated that the protein level of LDHA was up-regulated in OSCC tumor tissues (Figure 1F). The results of an analysis of the mRNA levels of LDHA in normal oral mucosal epithelial tissues and OSCC cell lines (SCC1, SCC25, HSC6, SCC15, and HSC3) indicated that the mRNA level of LDHA was also highly expressed in OSCC cells lines compared with that in normal oral mucosal epithelial tissues (Figure 1G). Next, 284 OSCC samples and 30 matched normal oral mucosal epithelial tissues in the TCGA database were compared; an up-regulation of LDHA mRNA levels in the OSCC samples was evident (Figure 1H). A survival analysis revealed a negative correlation between the high LDHA expression and overall survival rates; however, there was no statistical difference in the rates for disease-free survival and disease-specific survival (Figure 1I).

**Table 1:** Association between LDHA expression and clinicopathological features in patients with OSCC.

**Clinicopathologic** **features**	**No. of cases**	**LDHA expression**		***P*-value**
		**High**	**Low**	
**Gender**				
Male	58	35	23	0.0969
Female	31	13	18	
**Age**				
≥60	54	22	20	0.7813
<60	35	26	21	
**Differentiation**				
Well	42	16	26	0.0046^*^
Moderate + poor	47	32	15	
**T stage**				
T1-2	75	37	38	0.0439^*^
T3-4	14	11	3	
**TNM stage**				
I-II	60	29	31	0.1274
III-IV	29	19	10	
**N stage**				
N^−^	69	35	34	0.2594
N^+^	20	13	7	

*Statistical method: Fisher's exact test*.

* *Statistically significant: p < 0.05*.

*Proportion of positive cells (0–100%) and intensity of the cytoplasmic staining (0: no staining, 1: weak, 2: moderate, and 3: strong) were used to calculate the LDHA Histo-score. The score of each slice was determined using the staining index (proportion of positive cells × staining intensity) (H-score: 0–300). Tissues with an H-score >150 were classified as the high-expression group, whereas H-scores ≤ 150 were classified as the low-expression group*.

### Construction of a Weighted Correlation Network and the Target Module Identification

WGCNA was used to explore potential gene modules related to LDHA expression in the gene expression data of patients with OSCC. Seven abnormal samples were removed by establishing the connection screening threshold, and the remaining 277 samples were clustered with LDHA expression levels (Supplementary Figure 1A). The soft threshold was selected as β = 6 to obtain the neighboring and topology matrices, which caused 6,000 of the most variant genes to conform to the scale-free network (Supplementary Figure 1B). The topological overlap of the gene networks can be visualized using heatmaps (Supplementary Figure 1C). The clustering tree was divided into 11 modules using dynamic shearing (gray represents a gene not assigned to any module; the module's minimum gene number is 30), and the modules were merged according to the coefficient of dissimilarity of <0.2 (Supplementary Figures 1D,E). Supplementary Figures 1F,G illustrate the relationships between LDHA expression and the module eigengenes and the correlation heatmap of each module with LDHA expression. The module most significantly associated with LDHA expression (*r* = 0.39, *p* = 1 × 10^−11^) was the red module. By measuring the correlation between gene significance and module membership, the genes in the red module that were highly correlated with LDHA expression were often the most crucial elements of modules associated with LDHA expression (Supplementary Figure 1H).

### GO and KEGG Analysis for the Target Module

**Figure 2:**
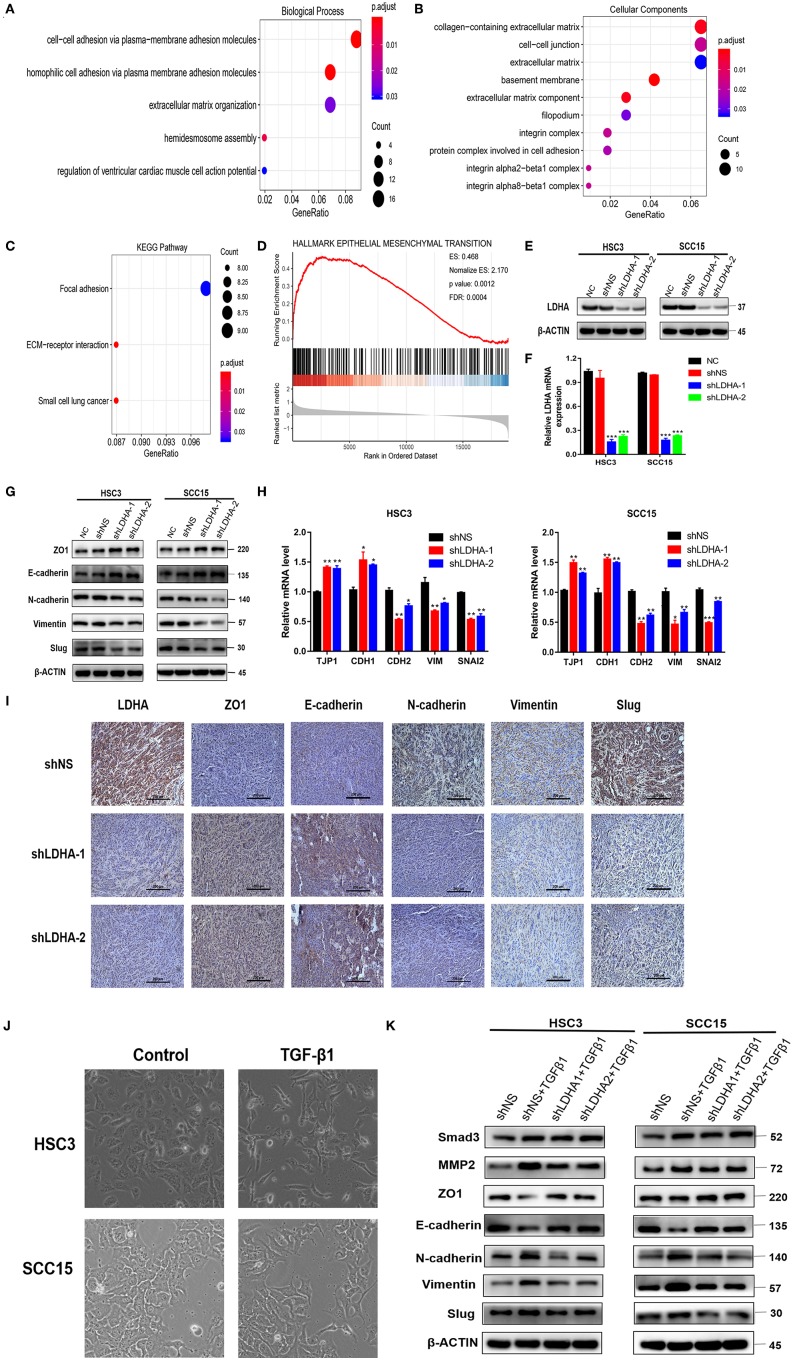
LDHA promotes malignant progression of OSCC by inducing EMT. **(A,B)** GO enrichment analysis of the target module genes, biological process analysis, and cellular component analysis. **(C)** KEGG enrichment analysis of the target module genes. **(D)** Epithelial-mesenchymal transition gene set with statistically significant differences in GSEA using Hallmark gene sets. **(E,F)** Western blot and RT-qPCR were used to detect the expression level of LDHA following transfection. **(G,H)** Western blot and RT-qPCR were used to detect the expression level of the EMT-related marker in stable LDHA-knockdown HSC3 and SCC15 cells. **(I)** Sections of tumor xenografts from shNS or shLDHA HSC3 cells subcutaneously injected nude mice were stained with LDHA, ZO1, E-cadherin, N-cadherin, Vimentin and Slug antibodies by IHC. Magnification at 200×. **(J)** HSC3 and SCC15 cells were treated with or without 10 ng/ml TGF-β for 48 h; the phenotypic changes of cells were recorded using microscopy. Magnification at 100×. **(K)** The stable LDHA-knockdown HSC3 and SCC15 cells treated with or without 10 ng/ml TGF-β for 48 h, protein expression was determined by Western blot analysis. The experiment was repeated three times; error bars indicate standard deviation. *p < 0.05, **p < 0.01, ***p < 0.001.

To further understand the gene function in the red module, this study applied the R package “clusterProfiler” for GO and KEGG analyses (Supplementary Data Sheet 1). The results of the GO analysis results revealed that the enrichment of genes in the biological process (BP) classification includes the cell–cell adhesion via plasma–membrane adhesion molecules, homophilic cell adhesion via plasma membrane adhesion molecules, hemidesmosome assembly, extracellular matrix (ECM) organization, and regulation of ventricular cardiac muscle cell action potential (Figure 2A). Genes in the red module were mainly enriched in the ECM region in the cellular component classification (Figure 2B). The results of KEGG analysis demonstrated that the genes were mainly enriched during ECM-receptor interaction, small cell lung cancer, focal adhesion (Figure 2C).

### LDHA Regulates EMT Progression in OSCC Cells

The R package “clusterProfiler” was used to perform GSEA in all the differentially expressed genes between the LDHA high-expression and low-expression groups in the OSCC samples (19). Statistically significant differences in the GSEA (*p*-value < 0.05, FDR < 0.05) were revealed using Hallmark gene sets in 39 enriched gene sets (Supplementary Data Sheet 2) (20). GSEA results showed that the LDHA high-expression group activated more EMT progression compared with the LDHA low-expression group (Figure 2D). Based on the GSEA and WGCNA results, it is assumed that LDHA may induce EMT to promote the malignant progression of OSCC by affecting cell adhesion and changing the components of ECM. Therefore, a Western blot and RT-qPCR was conducted on human HSC3 and SCC15 cells with stable LDHA knockdown (shLDHA-1 and shLDHA-2) in order to detect EMT related markers [ZO1 (TJP1), E-cadherin (CDH1), N-cadherin (CDH2), Vimentin (VIM), and Slug (SNAI2)] (Figures 2E,F). The result showed that LDHA expression was negatively correlated with the epithelial phenotype and positively correlated with the mesenchymal phenotype (Figures 2G,H). The same results were also evident in the immunohistochemistry of tumors in the shLDHA group compared with those in the shNS group (Figure 2I). By inducing EMT in OSCC cells treated with 10 ng/ml of TGF-β1 for 48 h, we found that TGF-β 1 induced downregulation of the epithelial phenotype marker and the upregulation of the mesenchymal phenotype marker were rescued by LDHA knockdown (Figures 2J,K). Collectively, these data confirmed that LDHA loss inhibited the EMT progression of OSCC cells.

**Figure 3:**
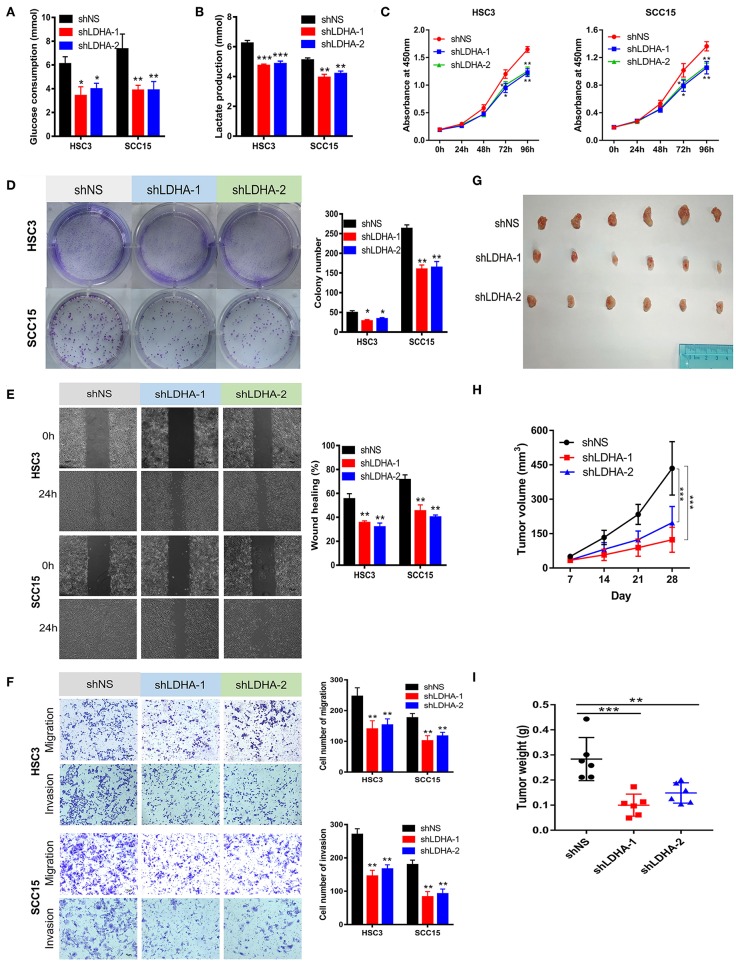
Silencing LDHA inhibits the aerobic glycolysis, proliferation, migration, and invasion of OSCC. **(A,B)** Glucose consumption and lactate production of LDHA knockdown HSC3 and SCC15 cells. **(C,D)** Proliferation of LDHA-suppressed HSC3 and SCC15 cells was detected using CCK-8 and colony formation assays. **(E)** Wound-healing of HSC3 and SCC15 cells after silencing LDHA was recorded and quantitatively analyzed. **(F)** Migration and invasion assay of LDHA knockdown HSC3 and SCC15 cells were photographed and measured; **(G)** Knockdown of LDHA inhibited HSC3 cells growth in NOD/SCID mice. **(H)** The volume of tumor xenografts in NOD/SCID mice were calculated weekly. **(I)** The tumor weight was measured on day 28. The experiment was repeated three times; error bars indicate standard deviation. *p < 0.05, **p < 0.01, ***p < 0.001.

### LDHA Silencing Inhibits Aerobic Glycolysis, Proliferation, Migration, and Invasion of OSCC Cells

This study revealed that LDHA promotes the EMT progression of OSCC and that lactate provides the necessary metabolic and biosynthetic raw materials for the growth of tumor cells (8). We then investigated whether LDHA was essential for the proliferation and metastasis of OSCC cells. The knockdown of LDHA decreased glucose consumption and lactate production in HSC3 and SCC15 cells (Figures 3A,B). Subsequently, we confirmed that the inhibition of LDHA suppressed the proliferation of HSC3 and SCC15 cells using theCCK-8 and colony formation assays (Figures 3C,D). Furthermore, wound-healing, migration, and invasion assays were utilized to detect the effect of LDHA on the migration and invasion of OSCC cells. As shown in Figures 3E,F, the knockdown of LDHA inhibited migration and invasion of HSC3 and SCC15 cells. We further examined the role of LDHA on OSCC tumor formation *in vivo* through subcutaneous injections of HSC3 cells into the inner thighs of NOD/SCID mice. The results revealed a significantly lower tumor volume and weight in the shLDHA group compared with the shNS group. This suggests that silencing LDHA can effectively inhibit OSCC tumor growth in mice (Figures 3G–I).

### Effects of Oxamate on the Biological Function of OSCC Cells

**Figure 4:**
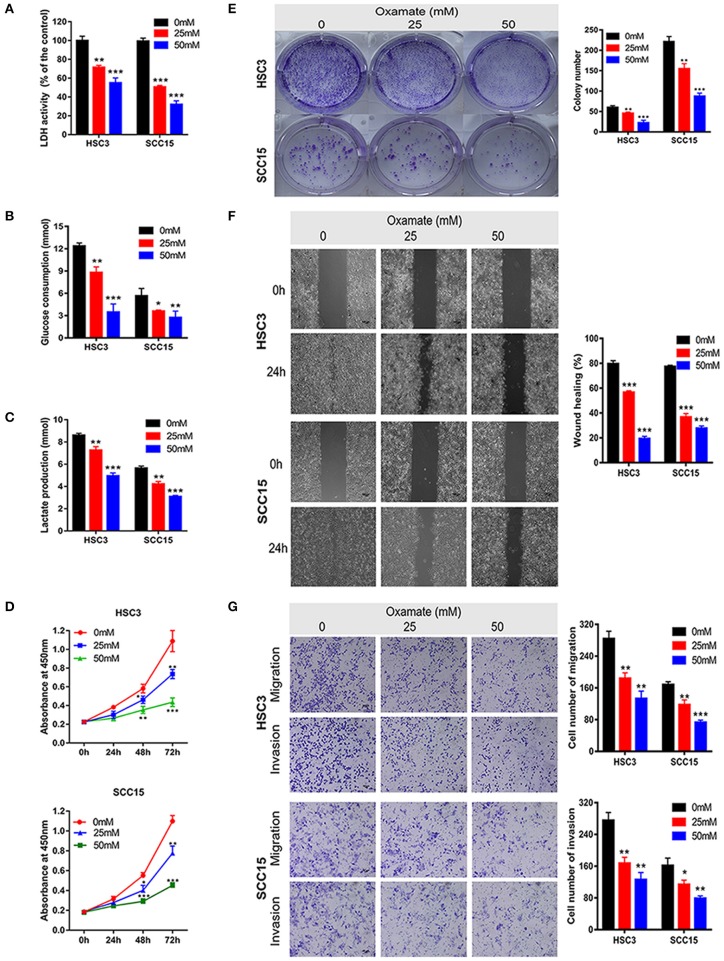
Effects of oxamate on the biological function of OSCC cells. **(A)** LDH activity of HSC3 and SCC15 cells was shown after exposure to oxamate for 24 h. **(B,C)** HSC3 and SCC15 cells were treated with oxamate for 24 h; glucose consumption and lactate production were detected. **(D,E)** Proliferation of HSC3 and SCC15 cells was detected using CCK-8 and colony formation assays after exposure to oxamate for 24 h. **(F)** Wound-healing of HSC3 and SCC15 cells after exposure to oxamate for 24 h was recorded and quantitatively analyzed. **(G)** HSC3 and SCC15 cells were treated with oxamate for 24 h; migration and invasion assays were photographed and measured. The experiment was repeated three times; error bars indicate standard deviation. *p < 0.05, **p < 0.01, ***p < 0.001.

To explore the potential efficacy of the targeted inhibition of LDHA, we used oxamate to inhibit the LDH activity to detect the biological function of OSCC cells (Figure 4A). As shown in Figures 4B,C, glucose consumption and lactate production were reduced in a dose-dependent manner by treating HSC3 and SCC15 cells with various concentrations of oxamate. Furthermore, oxamate also suppressed the proliferation, migration, and invasion of HSC3 and SCC15 cells in a dose-dependent manner (Figures 4D–G).

## Discussion

Elevated aerobic glycolysis remains the predominant feature of cancer metabolism and is deeply dependent on dysregulated metabolic enzymes (21, 22). The carcinogenic action of LDHA has been reported in several cancers, including pancreatic and gallbladder cancer, nasopharyngeal carcinoma, and breast cancer, and it promotes the malignant progression of cancer by increasing lactate production, accelerating glucose uptake, and regulating many cancer-associated molecules (9, 12, 23–25). However, the role of LDHA in the initiation and progression of OSCC is not yet fully understood. This study demonstrated that LDHA acted as an oncogene in OSCC to facilitate cell growth and metastasis both *in vitro* and *in vivo*. Treatment with oxamate reduced glucose consumption and lactate production in OSCC cells and inhibited cell proliferation, migration, and invasion. To determine the association between LDHA expression levels and OSCC progression, we performed WGCNA and GSEA using OSCC patients' gene expression data from the TCGA database. The results indicated that LDHA was closely associated with EMT and that silencing LDHA decreased the expression of EMT-related markers in OSCC cells. The present study revealed that LDHA promoted OSCC cell growth and metastasis by inducing EMT Progression.

As the last key enzyme in the glycolysis process, LDH plays an important role in the conversion of pyruvate to lactate, indicating its essential role in tumor metabolism. LDH is a homo- or heterotetramer composed of two subunits: LDHA (M) and LDHB (H); these are encoded by LDHA and LDHB, respectively, and exhibit different isoenzyme compositions in different tissues. The LDHA isoform is mainly expressed in skeletal muscle, which preferentially converts pyruvate into lactate, while the LDHB isoform is chiefly expressed in the heart and brain, where it preferentially converts lactate into pyruvate (26). In LDHA knockout human colon adenocarcinoma (LS174T) cells, the LDHB levels were upregulated under normoxic conditions; however, hypoxic conditions had no effect. However, the LDHB expression did not change in LDHA knockout mouse melanoma (B16-F10) cells, as analyzed using Western blotting (27). Additionally, the acquisition of ectopic fibroblast growth factor receptor 1 increased the stability of LDHA by tyrosine phosphorylation and reduced LDHB expression by supporting its promoter methylation, thereby promoting the conversion of prostate cancer cell metabolism from oxidative phosphorylation to aerobic glycolysis (28). In this study, LDHA was highly expressed in OSCC tissues and cells and was associated with poor prognosis in patients with OSCC. This indicates that LDHA acts as an oncogene in the context of OSCC.

To investigate the molecular mechanism by which LDHA promotes the progression of OSCC, we used WGCNA and GSEA to identify gene sets associated with LDHA expression using OSCC gene expression data provided by TCGA. For WGCNA, we constructed a weighted correlation network to identify the most significant module associated with LDHA expression, which contained 238 genes. According to the results of GO and KEGG enrichment analysis, key module genes were primarily enriched in cell adhesion and ECM function regulation. Recent studies reported that LDHA can promote the progression of renal cell carcinoma and bladder cancer by promoting EMT (29, 30). Moreover, lactate, the ultimate metabolite of aerobic glycolysis, led to an acidic tumor microenvironment that facilitated the degradation of ECM and the production of actin filaments (31). The degradation and remodeling of the ECM highly influences and controls both epithelial cell phenotype maintenance and EMT progression (32). Additionally, the mechanical properties and organization of the ECM are essential in regulating EMT (33). Thus, the results of GO and KEGG enrichment analysis revealed that EMT played a key role in the malignant progression of LDHA regulation of OSCC. GSEA indicated that the LDHA high-expression group activated more EMT gene sets compared with the LDHA low-expression group. This further demonstrates that LDHA regulates EMT in OSCC. We also examined the effect of LDHA on the expression of EMT-related markers. These results suggest that LDHA might induce EMT to promote the malignant progression of OSCC by affecting cell adhesion and changing the components of the ECM.

EMT is a key mechanism that promotes tumor migration and invasion (34, 35). Moreover, aerobic glycolysis remodels the ECM and facilitates EMT-inducing transcription factor expression by increasing the lactate content surrounding cancer cells and reducing the pH of the tumor microenvironment; these factors indicate that LDHA induces EMT and promotes cancer cell metastasis (8, 31, 33, 36). Additionally, the upregulation of LDHA enhances lactate production by aerobic glycolysis, which provides sufficient biomaterials and energy for the growth and metabolism of cancer cells. In recent years, numerous studies have reported that LDHA knockdown could suppress the growth and metastasis of cancer cells (9, 12, 25). This evidence suggests that LDHA promotes diverse malignant biocharacteristics. The results of the present study demonstrated that LDHA knockdown reduced glucose consumption and lactate production in OSCC cells and suppressed cell proliferation, migration, and invasion *in vitro*. Meanwhile, silencing LDHA inhibited the growth of OSCC cells *in vitro*. Results of comprehensive experiments conducted both *in vitro* and *in vivo*, LDHA exerted a cancer-promoting effect on the progression of OSCC by regulating cell glycolysis.

The catalytic substrate of LDHA is competitively inhibited by oxamate, a pyruvate analog, and its effect has been identified in various cell lines (15, 37–39). A study by Zhao et al. (15) demonstrated that oxamate inhibited the activity of gastric cancer cells in a dose- and time-dependent manner. El-Sisi et al. (37) documented that the combination of oxamate and Taxol reduced levels of proinflammatory mediators, such as TNF-α, IL-17, MDA and ATP, in solid Ehrlich carcinomas in mice and facilitated apoptosis and anti-angiogenic effects compared with paclitaxel monotherapy. In the present study, oxamate was shown to inhibit OSCC cell glycolysis, proliferation, and metastasis in a dose-dependent manner. However, owing to limited membrane permeability, the effective dose of oxamate administered to cancer cells *in vitro* is too high relative to *in vivo* administration, which limits its potential for clinical application. Currently, effective inhibitors of LDHA are under development, including Gossypol (40), FX11 (41), Quinoline 3-sulfonamides (42), NHI (43), Galloflavin (44). Gossypol has demonstrated clinical efficacy in metastatic adrenal cancer and recurrent adult malignant gliomas (45, 46). Developing specific and effective LDHA small molecule inhibitors will be a more appropriate therapeutic strategy for OSCC.

In summary, the present study confirmed the role of LDHA as an oncogene during OSCC progression. Compelling *in vitro* and *in vivo* evidence demonstrates that LDHA can promote the proliferation, migration, and invasion of OSCC cells by facilitating glycolysis and EMT progression. These findings provide strategies for silencing or deactivating LDHA in carcinoma tissues and may be a new therapeutic target for OSCC.

## Data Availability Statement

All datasets generated for this study are included in the article/Supplementary Material.

## Ethics Statement

The studies involving human participants were reviewed and approved by The ethics committee of the Hospital of Stomatology, Sun Yat-sen University. The patients/participants provided their written informed consent to participate in this study. The animal study was reviewed and approved by Institutional Animal Care and Use Committee of Sun Yat-sen University, Guangzhou, China.

## Author Contributions

HC and JH conceived and designed the experiments. HC, JL, YZha, and YL performed the experiments. HC, JL, YZhu, and CW collected and analyzed the data. HC wrote the original manuscript. CW and JH reviewed and edited the manuscript.

### Conflict of Interest

The authors declare that the research was conducted in the absence of any commercial or financial relationships that could be construed as a potential conflict of interest.

## Supplementary Material

Supplementary Data Sheet 1 Genes of red module.

Supplementary Data Sheet 2 Enriched gene sets of GSEA.

Supplementary Figure 1 Construction of weighted correlation network and target module identification. **(A)** Clustering dendrogram of OSCC samples and LDHA expression after removing outliers. **(B)** Analysis of network topology for various soft-thresholding powers. **(C)** The network heatmap plot depicts the topological overlap matrix among all genes in the analysis. The gene dendrogram and module assignment are also shown along the left side and at the top. A light color represents low overlap and a progressively darker red color represents higher overlap. **(D)** Clustering of modules' eigengenes; modules below the red line indicate correlation >0.8; these modules will be merged. **(E)** Cluster dendrogram of genes, with dissimilarity based on topological overlap together with assigned merged module colors and original module colors. Each color is assigned to one module (gray represents unassigned genes). **(F)** Visualization of the eigengene adjacency heatmap representing the relationships among the modules and LDHA expression. **(G)** Correlation values and p-value for module–LDHA relationships. **(H)** Scatterplot of the correlation between GS for LDHA and module membership in the red module.

Supplementary Figure 2 Original images of western blot.

## References

[1] Chi AC Day TA Neville BW. Oral cavity and oropharyngeal squamous cell carcinoma–an update. CA Cancer J Clin. (2015) 65:401–21. 10.3322/caac.21293 26215712

[2] Thomson PJ. Perspectives on oral squamous cell carcinoma prevention - proliferation, position, progression and prediction. J Oral Pathol Med. (2018) 47:803–7. 10.1111/jop.12733 29752860

[3] WARBURG O. On the origin of cancer cells. Science. (1956) 123:309–14. 10.1126/science.123.3191.309 13298683

[4] Chen G Zhang Y Liang J Li W Zhu Y Zhang M. Deregulation of hexokinase II is associated with glycolysis, autophagy, and the epithelial-mesenchymal transition in tongue squamous cell carcinoma under hypoxia. Biomed Res Int. (2018) 2018:8480762. 10.1155/2018/8480762 29682563 PMC5841093

[5] Chen G Liu H Zhang Y Liang J Zhu Y Zhang M. Silencing PFKP inhibits starvation-induced autophagy, glycolysis, and epithelial mesenchymal transition in oral squamous cell carcinoma. Exp Cell Res. (2018) 370:46–57. 10.1016/j.yexcr.2018.06.007 29894707

[6] San-Millan I Brooks GA. Reexamining cancer metabolism: lactate production for carcinogenesis could be the purpose and explanation of the Warburg Effect. Carcinogenesis. (2017) 38:119–33. 10.1093/carcin/bgw127 27993896 PMC5862360

[7] Pennington Z Goodwin ML Westbroek EM Cottrill E Ahmed AK Sciubba DM. Lactate and cancer: spinal metastases and potential therapeutic targets (part 2). Ann Transl Med. (2019) 7:221. 10.21037/atm.2019.01.85 31297386 PMC6595206

[8] Ippolito L Morandi A Giannoni E Chiarugi P. Lactate: a metabolic driver in the tumour landscape. Trends Biochem Sci. (2019) 44:153–66. 10.1016/j.tibs.2018.10.011 30473428

[9] Cui J Shi M Xie D Wei D Jia Z Zheng S. FOXM1 promotes the warburg effect and pancreatic cancer progression via transactivation of LDHA expression. Clin Cancer Res. (2014) 20:2595–606. 10.1158/1078-0432.CCR-13-2407 24634381 PMC4024335

[10] Jiang W Zhou F Li N Li Q Wang L. FOXM1-LDHA signaling promoted gastric cancer glycolytic phenotype and progression. Int J Clin Exp Pathol. (2015) 8:6756–63. 26261559 PMC4525893

[11] Su Y Yu QH Wang XY Yu LP Wang ZF Cao YC. JMJD2A promotes the Warburg effect and nasopharyngeal carcinoma progression by transactivating LDHA expression. BMC Cancer. (2017) 17:477. 10.1186/s12885-017-3473-4 28693517 PMC5504777

[12] He Y Chen X Yu Y Li J Hu Q Xue C. LDHA is a direct target of miR-30d-5p and contributes to aggressive progression of gallbladder carcinoma. Mol Carcinog. (2018) 57:772–83. 10.1002/mc.22799 29569755

[13] Zhang K Mu L Ding MC Xu R Ding ZJ Liang J. NFkappaB mediated elevation of KCNJ11 promotes tumor progression of hepatocellular carcinoma through interaction of lactate dehydrogenase A. Biochem Biophys Res Commun. (2018) 495:246–53. 10.1016/j.bbrc.2017.11.011 29108994

[14] Zhou Y Niu W Luo Y Li H Xie Y Wang H. p53/Lactate dehydrogenase A axis negatively regulates aerobic glycolysis and tumor progression in breast cancer expressing wild-type p53. Cancer Sci. (2019) 110:939–49. 10.1111/cas.13928 30618169 PMC6398928

[15] Zhao Z Han F Yang S Wu J Zhan W. Oxamate-mediated inhibition of lactate dehydrogenase induces protective autophagy in gastric cancer cells: involvement of the Akt-mTOR signaling pathway. Cancer Lett. (2015) 358:17–26. 10.1016/j.canlet.2014.11.046 25524555

[16] Langfelder P Horvath S. WGCNA: an R package for weighted correlation network analysis. BMC Bioinform. (2008) 9:559. 10.1186/1471-2105-9-559 19114008 PMC2631488

[17] Rarnanathan A Wang C Schreiber SL Perturbational profiling of a cell-line model of tumorigenesis by using metabolic measurements. Proc Natl Acad Sci USA. (2005) 102:5992–7. 10.1073/pnas.0502267102 15840712 PMC1087961

[18] Goldman M Craft B Hastie M Repečka K Kamath A McDade F The UCSC Xena Platform for cancer genomics data visualization and interpretation. bioRxiv [Preprint]. (2018). 10.1101/326470

[19] Yu G Wang LG Han Y He QY. clusterProfiler: an R package for comparing biological themes among gene clusters. OMICS. (2012) 16:284–7. 10.1089/omi.2011.0118 22455463 PMC3339379

[20] Liberzon A Birger C Thorvaldsdottir H Ghandi M Mesirov JP Tamayo P. The molecular signatures database (MSigDB) hallmark gene set collection. Cell Syst. (2015) 1:417–25. 10.1016/j.cels.2015.12.004 26771021 PMC4707969

[21] Feng Y Xiong Y Qiao T Li X Jia L Han Y. Lactate dehydrogenase A: a key player in carcinogenesis and potential target in cancer therapy. Cancer Med. (2018) 7:6124–36. 10.1002/cam4.1820 30403008 PMC6308051

[22] Jiang B. Aerobic glycolysis and high level of lactate in cancer metabolism and microenvironment. Genes Dis. (2017) 4:25–7. 10.1016/j.gendis.2017.02.003 30258905 PMC6136593

[23] Zhai X Yang Y Wan J Zhu R Wu Y. Inhibition of LDH-A by oxamate induces G2/M arrest, apoptosis and increases radiosensitivity in nasopharyngeal carcinoma cells. Oncol Rep. (2013) 30:2983–91. 10.3892/or.2013.2735 24064966

[24] Xiao X Huang X Ye F Chen B Song C Wen J. The miR-34a-LDHA axis regulates glucose metabolism and tumor growth in breast cancer. Sci Rep. (2016) 6:21735. 10.1038/srep21735 26902416 PMC4763192

[25] Pathria G Scott DA Feng Y Sang Lee J Fujita Y Zhang G. Targeting the warburg effect via LDHA inhibition engages ATF4 signaling for cancer cell survival. EMBO J. (2018) 37:e99735. 10.15252/embj.201899735 30209241 PMC6187221

[26] Markert CL Shaklee JB Whitt GS. Evolution of a gene. Multiple genes for LDH isozymes provide a model of the evolution of gene structure, function and regulation. Science. (1975) 189:102–14. 10.1126/science.1138367 1138367

[27] Zdralevic M Brand A Di Ianni L Dettmer K Reinders J Singer K. Double genetic disruption of lactate dehydrogenases A and B is required to ablate the Warburg effect restricting tumor growth to oxidative metabolism. J Biol Chem. (2018) 293:15947–61. 10.1074/jbc.RA118.004180 30158244 PMC6187639

[28] Liu J Chen G Liu Z Liu S Cai Z You P. Aberrant FGFR tyrosine kinase signaling enhances the warburg effect by reprogramming LDH isoform expression and activity in prostate cancer. Cancer Res. (2018) 78:4459–70. 10.1158/0008-5472.CAN-17-3226 29891507 PMC6095720

[29] Zhao J Huang X Xu Z Dai J He H Zhu Y. LDHA promotes tumor metastasis by facilitating epithelialmesenchymal transition in renal cell carcinoma. Mol Med Rep. (2017) 16:8335–44. 10.3892/mmr.2017.7637 28983605

[30] Jiang F Ma S Xue Y Hou J Zhang Y. LDH-A promotes malignant progression via activation of epithelial-to-mesenchymal transition and conferring stemness in muscle-invasive bladder cancer. Biochem Biophys Res Commun. (2016) 469:985–92. 10.1016/j.bbrc.2015.12.078 26721441

[31] Webb BA Chimenti M Jacobson MP Barber DL. Dysregulated pH: a perfect storm for cancer progression. Nat Rev Cancer. (2011) 11:671–7. 10.1038/nrc3110 21833026

[32] Tzanakakis G Kavasi RM Voudouri K Berdiaki A Spyridaki I Tsatsakis A. Role of the extracellular matrix in cancer-associated epithelial to mesenchymal transition phenomenon. Dev Dyn. (2018) 247:368–81. 10.1002/dvdy.24557 28758355

[33] Scott LE Weinberg SH Lemmon CA. Mechanochemical signaling of the extracellular matrix in epithelial-mesenchymal transition. Front Cell Dev Biol. (2019) 7:135. 10.3389/fcell.2019.00135 31380370 PMC6658819

[34] Thiery JP Acloque H Huang RY Nieto MA. Epithelial-mesenchymal transitions in development and disease. Cell. (2009) 139:871–90. 10.1016/j.cell.2009.11.007 19945376

[35] Brabletz T Kalluri R Nieto MA Weinberg RA. EMT in cancer. Nat Rev Cancer. (2018) 18:128–34. 10.1038/nrc.2017.118 29326430

[36] Sciacovelli M Frezza C. Metabolic reprogramming and epithelial-to-mesenchymal transition in cancer. FEBS J. (2017) 284:3132–44. 10.1111/febs.14090 28444969 PMC6049610

[37] El-Sisi AE Sokar SS Abu-Risha SE El-Mahrouk SR. Oxamate potentiates taxol chemotherapeutic efficacy in experimentally-induced solid ehrlich carcinoma (SEC) in mice. Biomed Pharmacother. (2017) 95:1565–73. 10.1016/j.biopha.2017.09.090 28950656

[38] Manerba M Govoni M Manet I Leale A Comparone A Di Stefano G. Metabolic activation triggered by cAMP in MCF-7 cells generates lethal vulnerability to combined oxamate/etomoxir. Biochim Biophys Acta Gen Subj. (2019) 1863:1177–86. 10.1016/j.bbagen.2019.04.008 30981740

[39] Valvona CJ Fillmore HL Oxamate, but not selective targeting of LDH-A, inhibits medulloblastoma cell glycolysis, growth and motility. Brain Sci. (2018) 8:E56 10.3390/brainsci8040056 29601482 PMC5924392

[40] Rani R Kumar V. Recent update on human lactate dehydrogenase enzyme 5 (hLDH5) inhibitors: a promising approach for cancer chemotherapy. J Med Chem. (2016) 59:487–96. 10.1021/acs.jmedchem.5b00168 26340601

[41] Le A Cooper CR Gouw AM Dinavahi R Maitra A Deck LM. Inhibition of lactate dehydrogenase A induces oxidative stress and inhibits tumor progression. Proc Natl Acad Sci USA. (2010) 107:2037–42. 10.1073/pnas.0914433107 20133848 PMC2836706

[42] Billiard J Dennison JB Briand J Annan RS Chai D Colon M. Quinoline 3-sulfonamides inhibit lactate dehydrogenase A and reverse aerobic glycolysis in cancer cells. Cancer Metab. (2013) 1:19. 10.1186/2049-3002-1-19 24280423 PMC4178217

[43] Granchi C Roy S Giacomelli C Macchia M Tuccinardi T Martinelli A. Discovery of N-hydroxyindole-based inhibitors of human lactate dehydrogenase isoform A (LDH-A) as starvation agents against cancer cells. J Med Chem. (2011) 54:1599–612. 10.1021/jm101007q 21332213

[44] Manerba M Vettraino M Fiume L Di Stefano G Sartini A Giacomini E. Galloflavin (CAS 568–80-9): a novel inhibitor of lactate dehydrogenase. ChemMedChem. (2012) 7:311–7. 10.1002/cmdc.201100471 22052811

[45] Flack MR Pyle RG Mullen NM Lorenzo B Wu YW Knazek RA. Oral gossypol in the treatment of metastatic adrenal cancer. J Clin Endocrinol Metab. (1993) 76:1019–24. 10.1210/jcem.76.4.8473376 8473376

[46] Bushunow P Reidenberg MM Wasenko J Winfield J Lorenzo B Lemke S. Gossypol treatment of recurrent adult malignant gliomas. J Neurooncol. (1999) 43:79–86. 10.1023/A:1006267902186 10448875

