# Supplementary material for: LDHA Promotes Oral Squamous Cell Carcinoma Progression Through Facilitating Glycolysis and Epithelial–Mesenchymal Transition
Source: Front Oncol. 2019 Dec 19;9:1446. doi: 10.3389/fonc.2019.01446 (PMC6930919; doi:10.3389/fonc.2019.01446)
Supplement: Supplementary Figure 1 — Construction of weighted correlation network and target module identification. (A) Clustering dendrogram of OSCC samples and LDHA expression after removing outliers. (B) Analysis of network topology for various soft-thresholding powers. (C) The network heatmap plot depicts the topological overlap matrix among all genes in the analysis. The gene dendrogram and module assignment are also shown along the left side and at the top. A light color represents low overlap and a progressively darker red color represents higher overlap. (D) Clustering of modules' eigengenes; modules below the red line indicate correlation >0.8; these modules will be merged. (E) Cluster dendrogram of genes, with dissimilarity based on topological overlap together with assigned merged module colors and original module colors. Each color is assigned to one module (gray represents unassigned genes). (F) Visualization of the eigengene adjacency heatmap representing the relationships among the modules and LDHA expression. (G) Correlation values and p-value for module–LDHA relationships. (H) Scatterplot of the correlation between GS for LDHA and module membership in the red module. [file Table_3.DOCX]

Supplementary Material

##
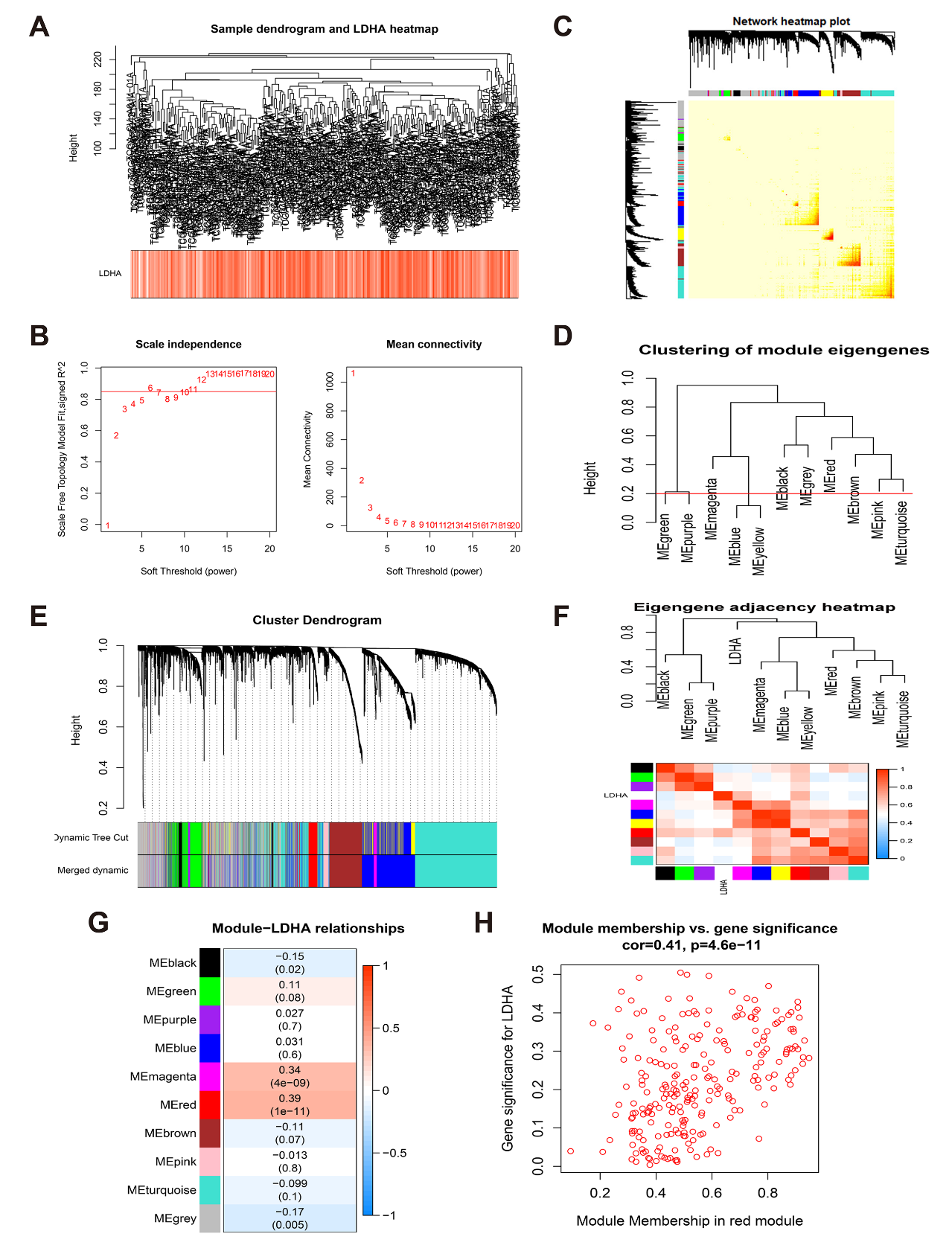
Supplementary Figures

**Supplementary Figure 1.** Construction of weighted correlation network and target module identification. (A) Clustering dendrogram of OSCC samples and LDHA expression after removing outliers. (B) Analysis of network topology for various soft-thresholding powers. (C) The network heatmap plot depicts the topological overlap matrix among all genes in the analysis. The gene dendrogram and module assignment are also shown along the left side and at the top. A light color represents low overlap and a progressively darker red color represents higher overlap. (D) Clustering of modules’ eigengenes; modules below the red line indicate correlation >0.8; these modules will be merged. (E) Cluster dendrogram of genes, with dissimilarity based on topological overlap together with assigned merged module colors and original module colors. Each color is assigned to one module (gray represents unassigned genes). (F) Visualization of the eigengene adjacency heatmap representing the relationships among the modules and LDHA expression. (G) Correlation values and p-value for module–LDHA relationships. (H) Scatterplot of the correlation between GS for LDHA and module membership in the red module.


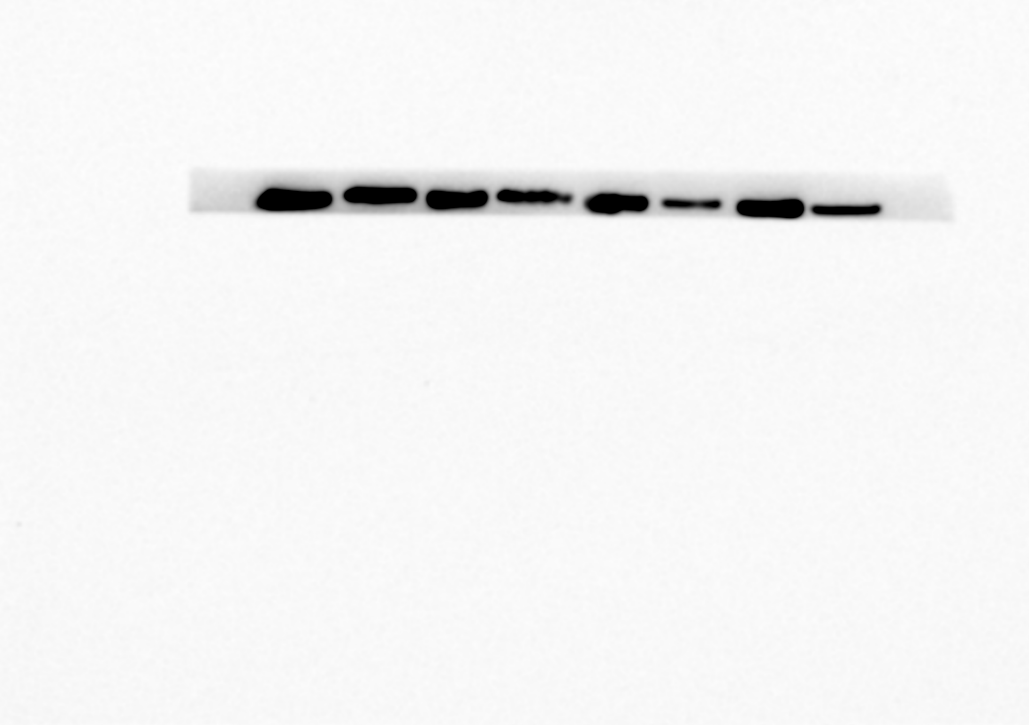

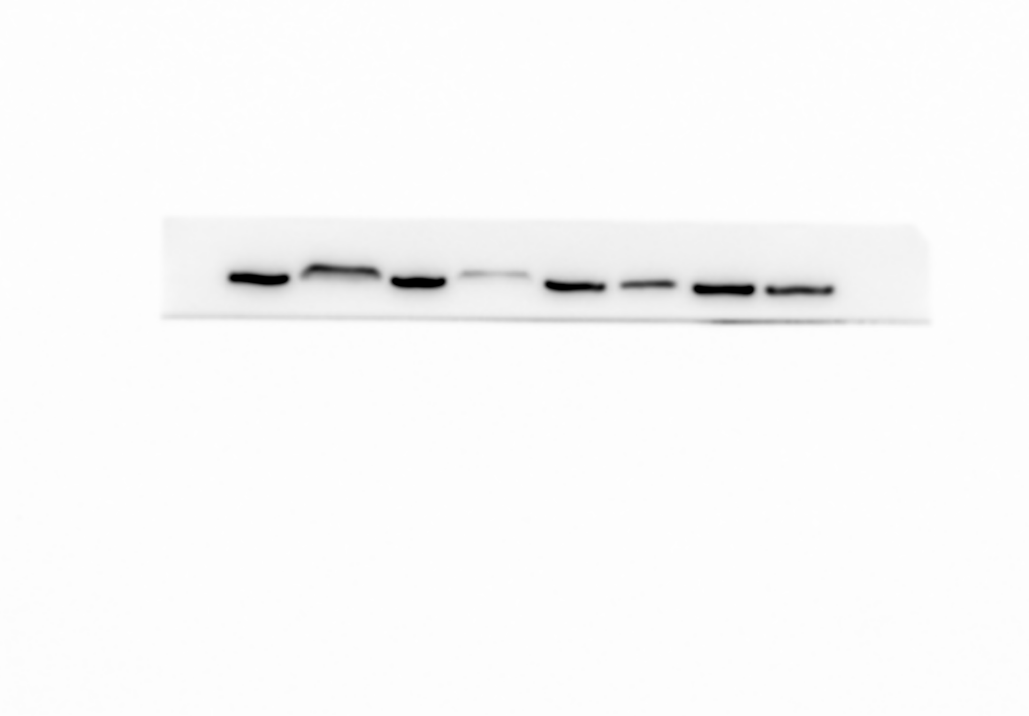

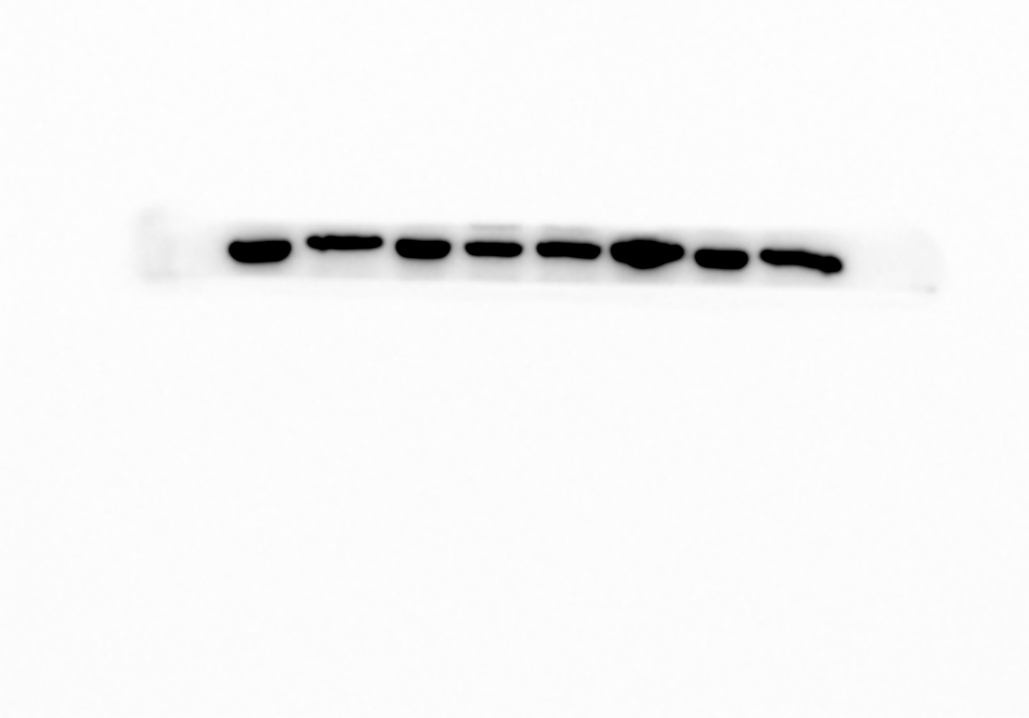

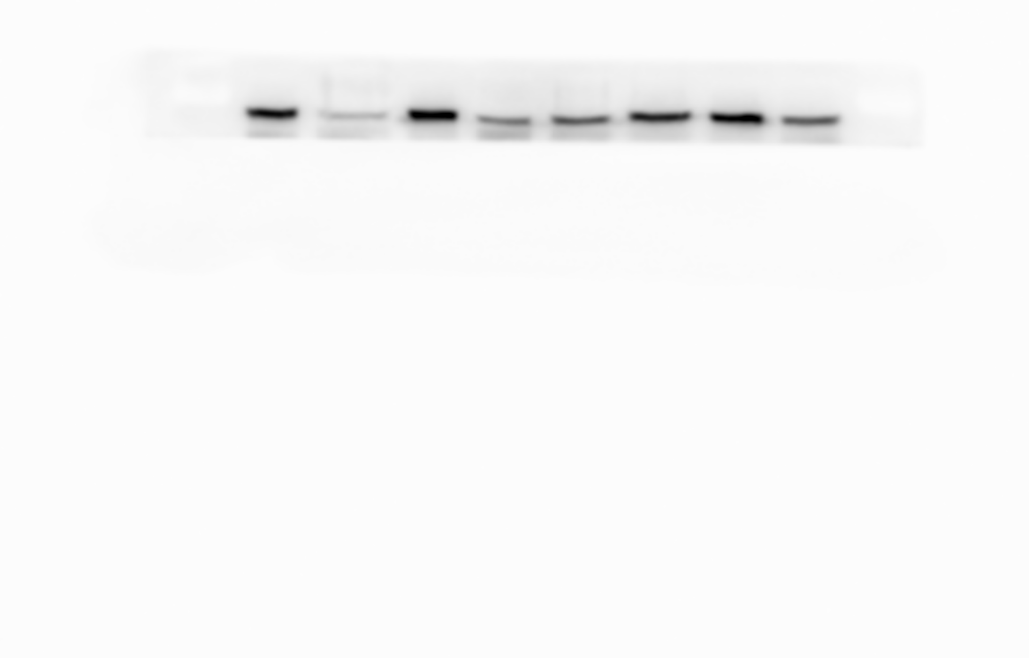

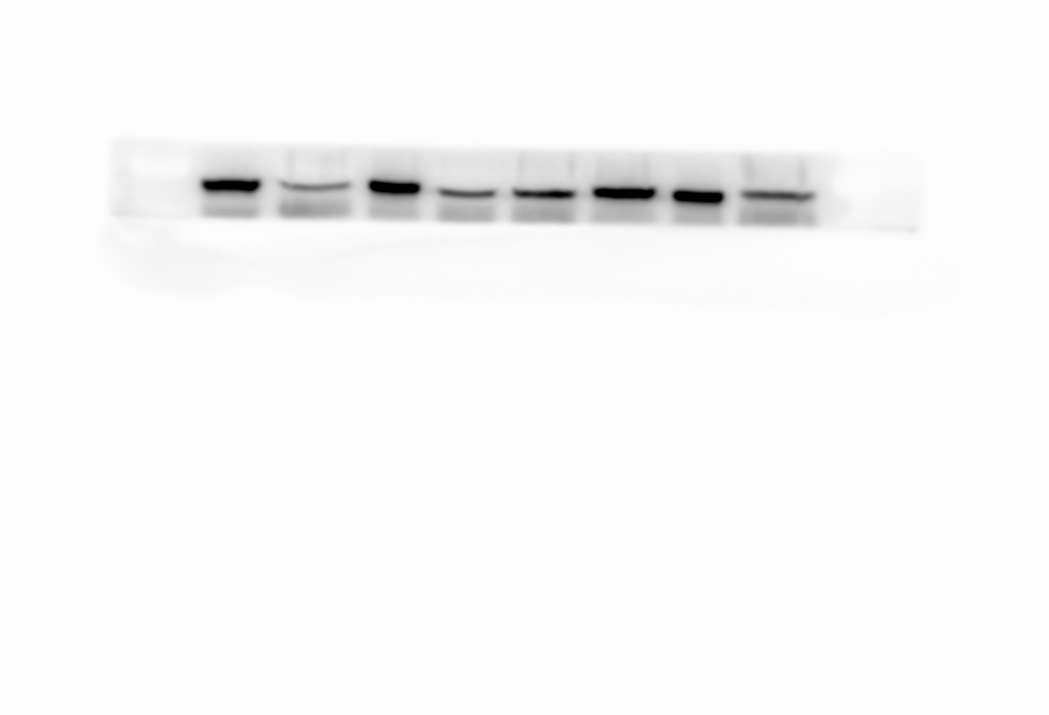

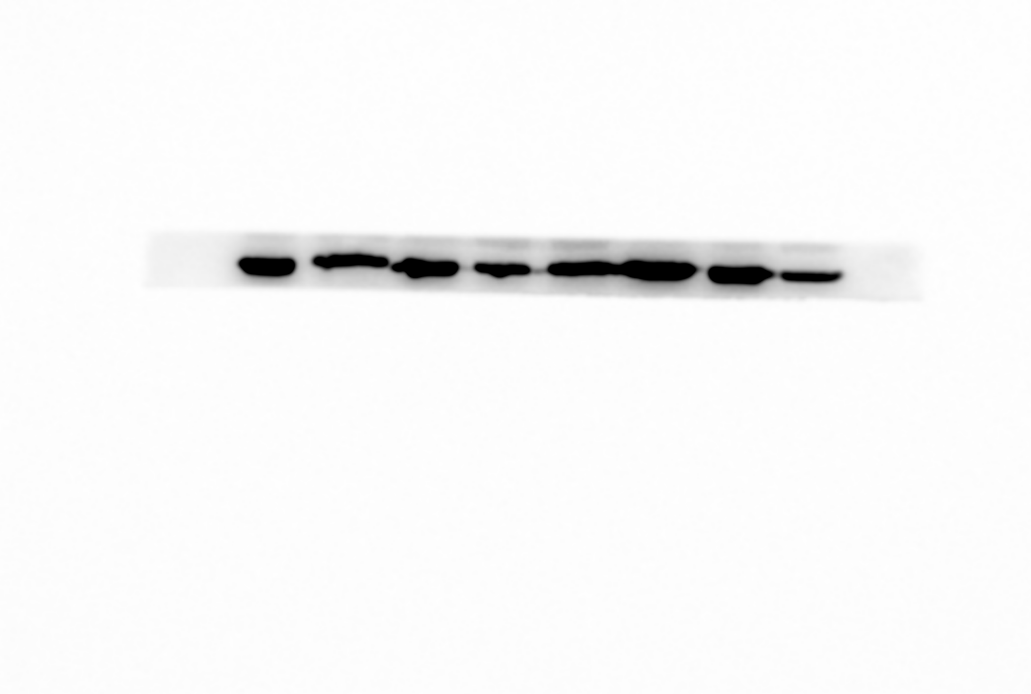

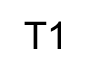

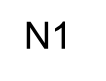

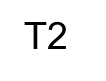

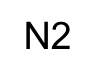

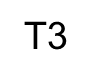

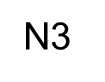

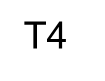

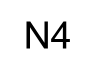

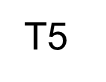

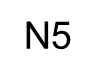

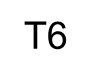

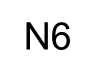

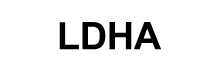

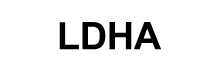

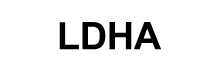

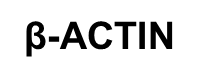

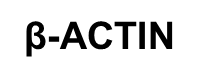

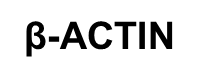


**Figure 1F**

**Figure 2E**


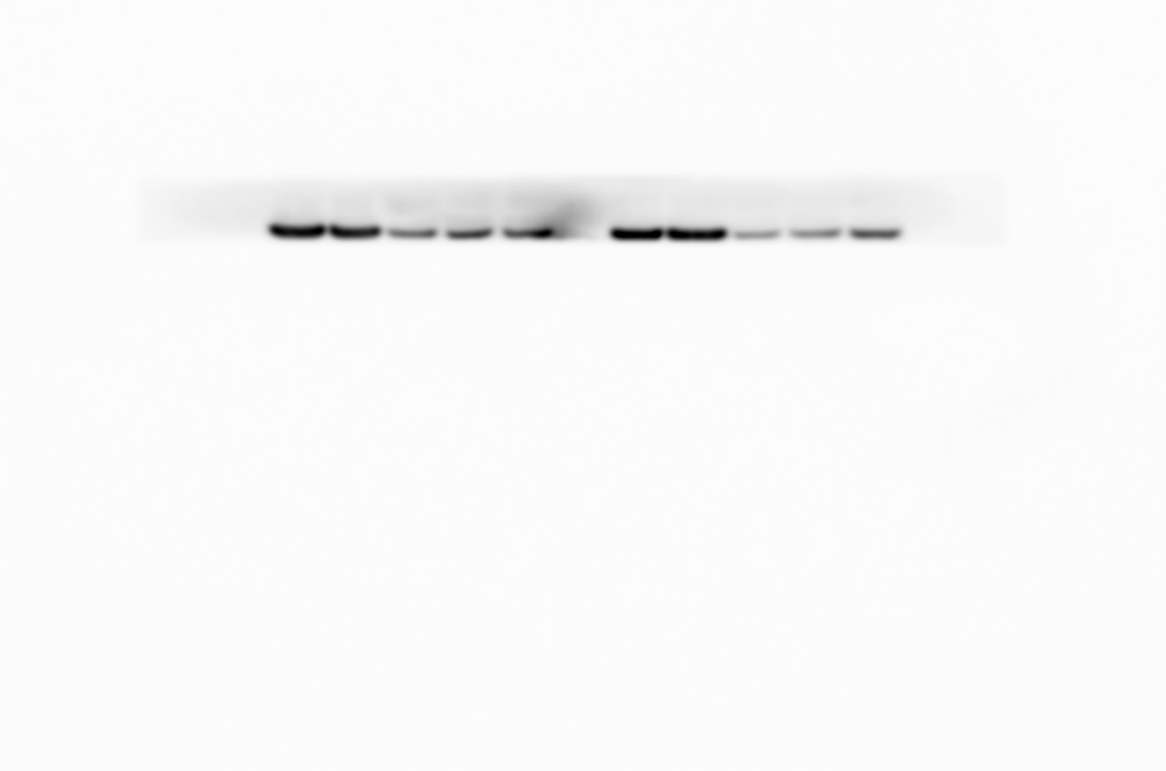


**HSC3**


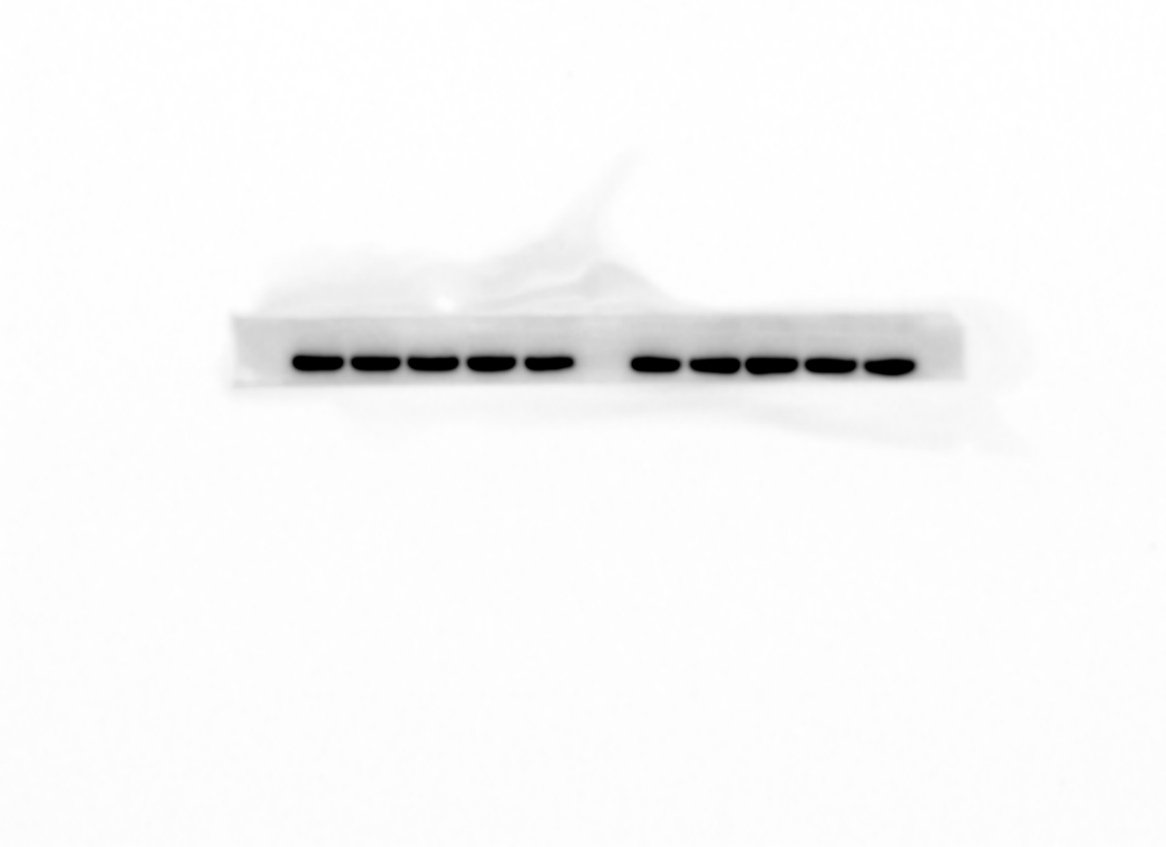


**SCC15**


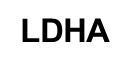

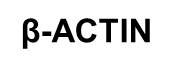


**Figure 2G**


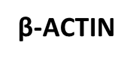

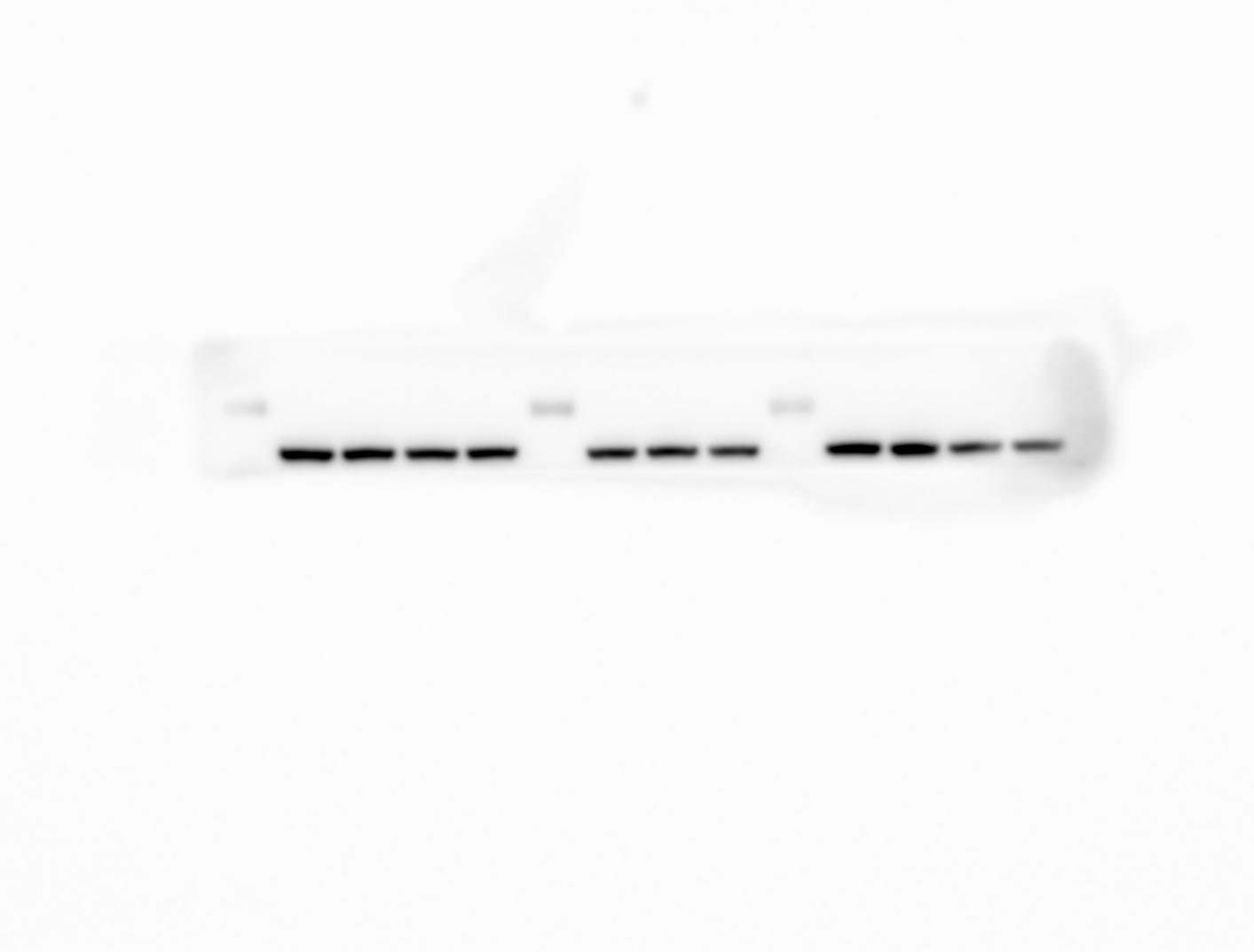

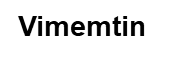

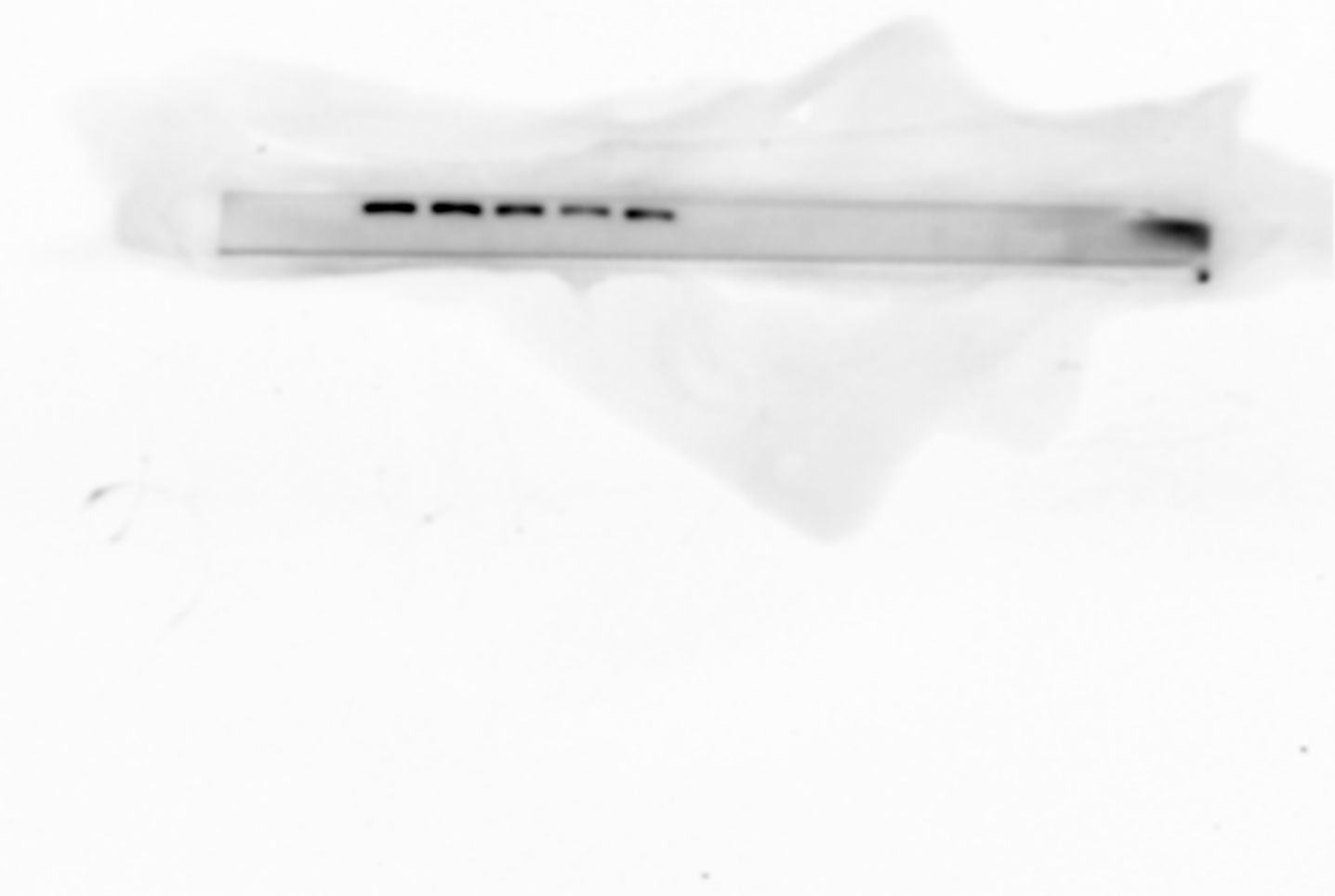

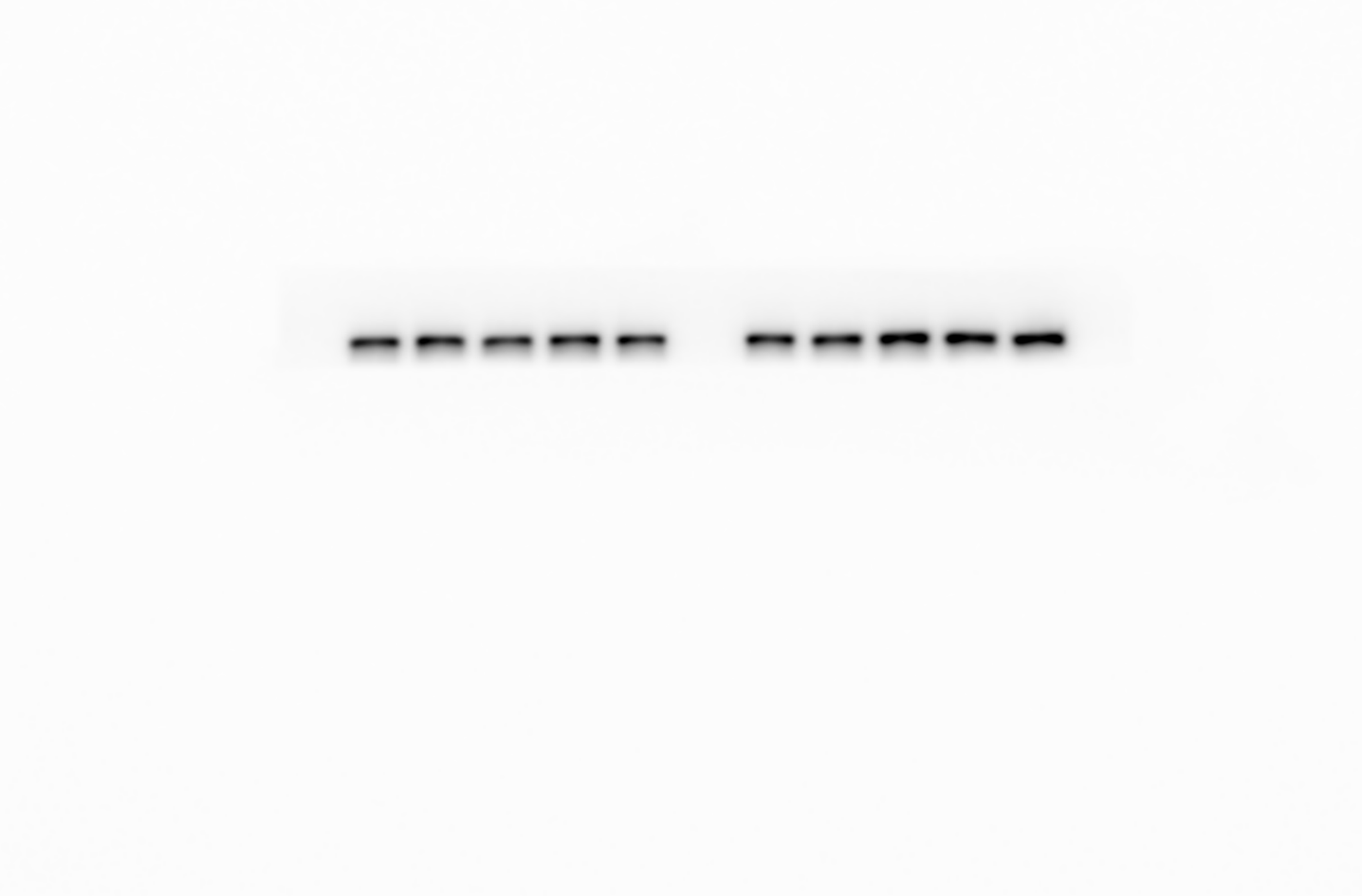

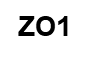

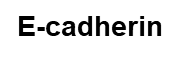

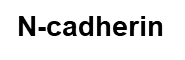

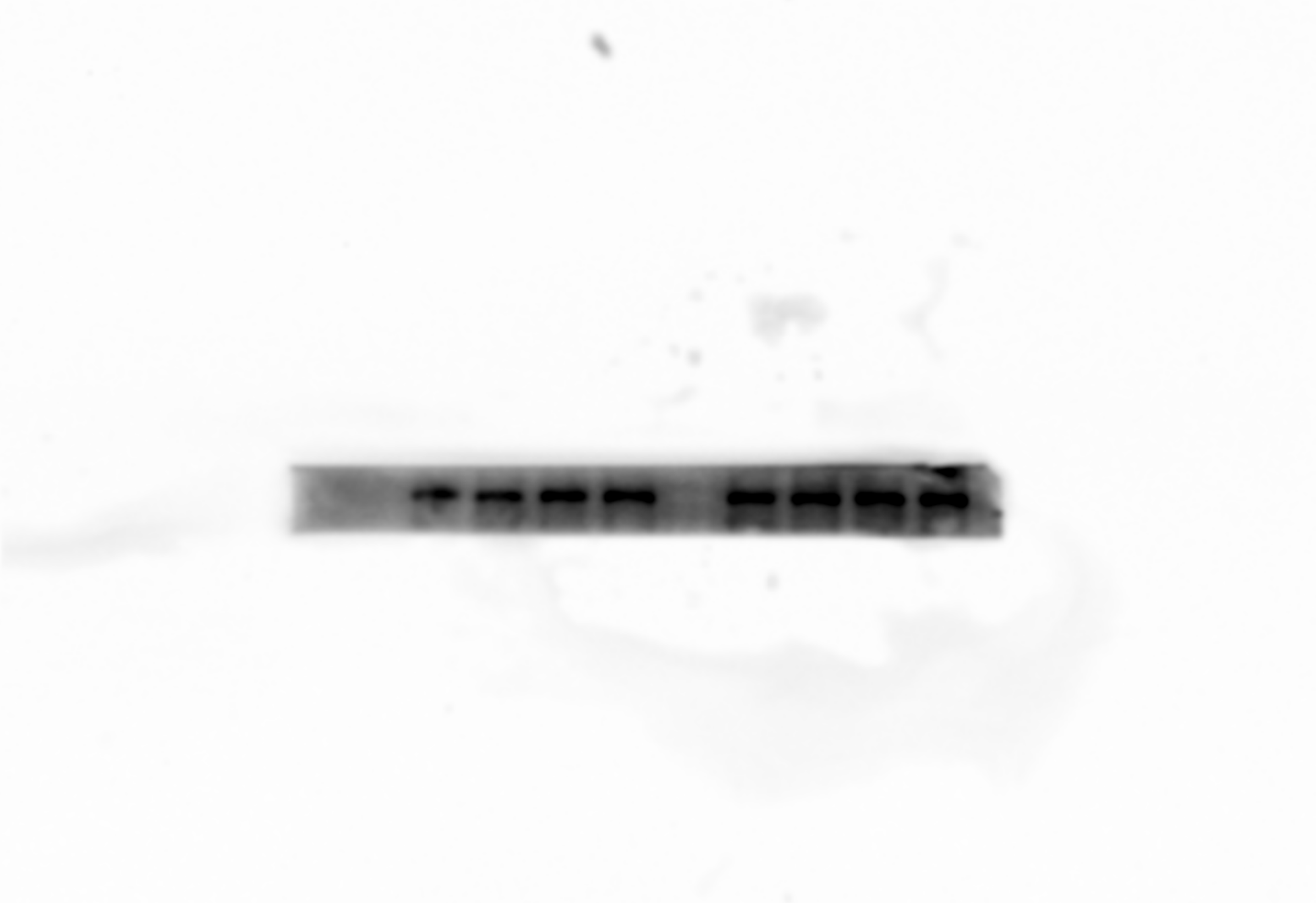

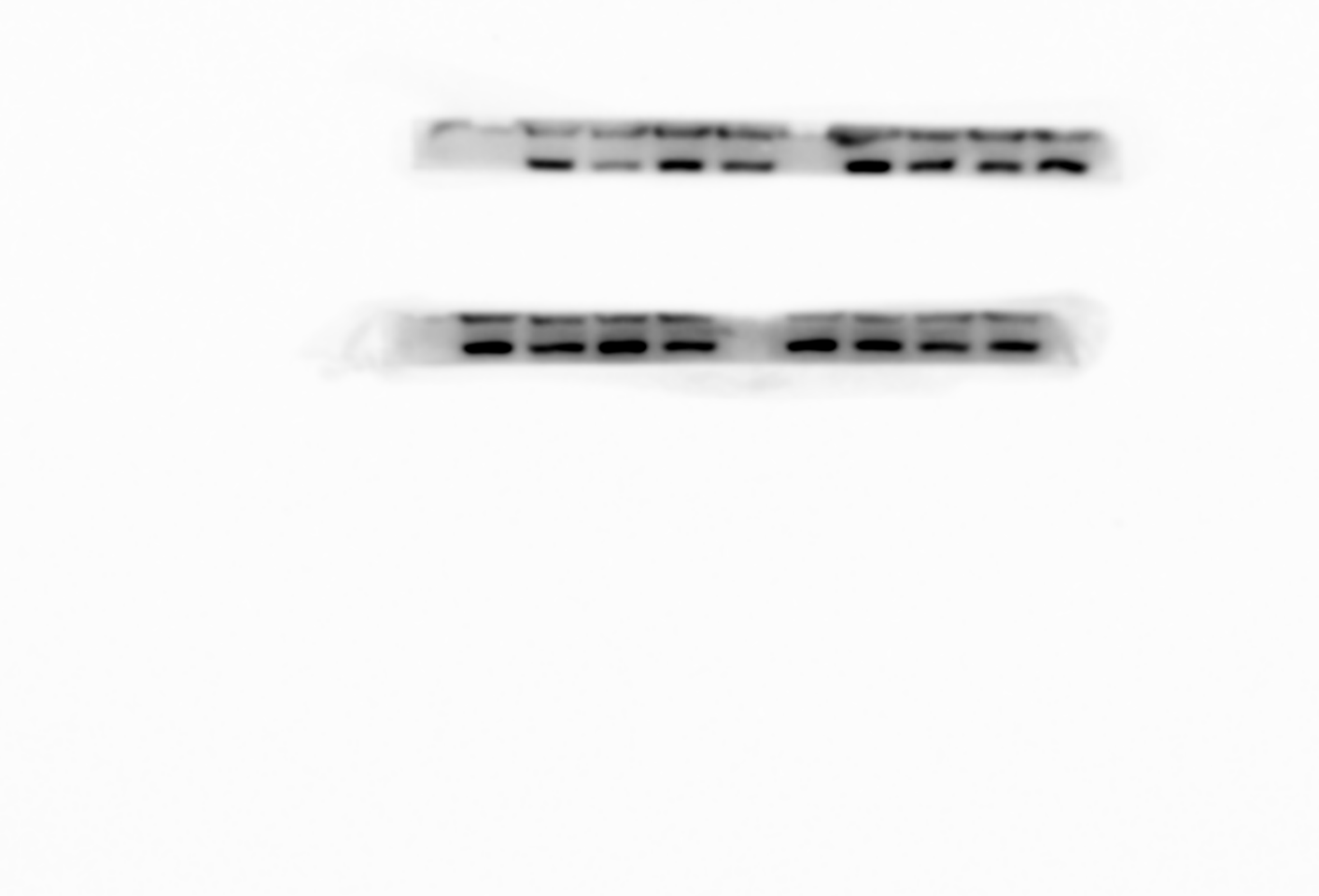


**SCC15**


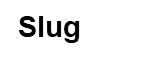

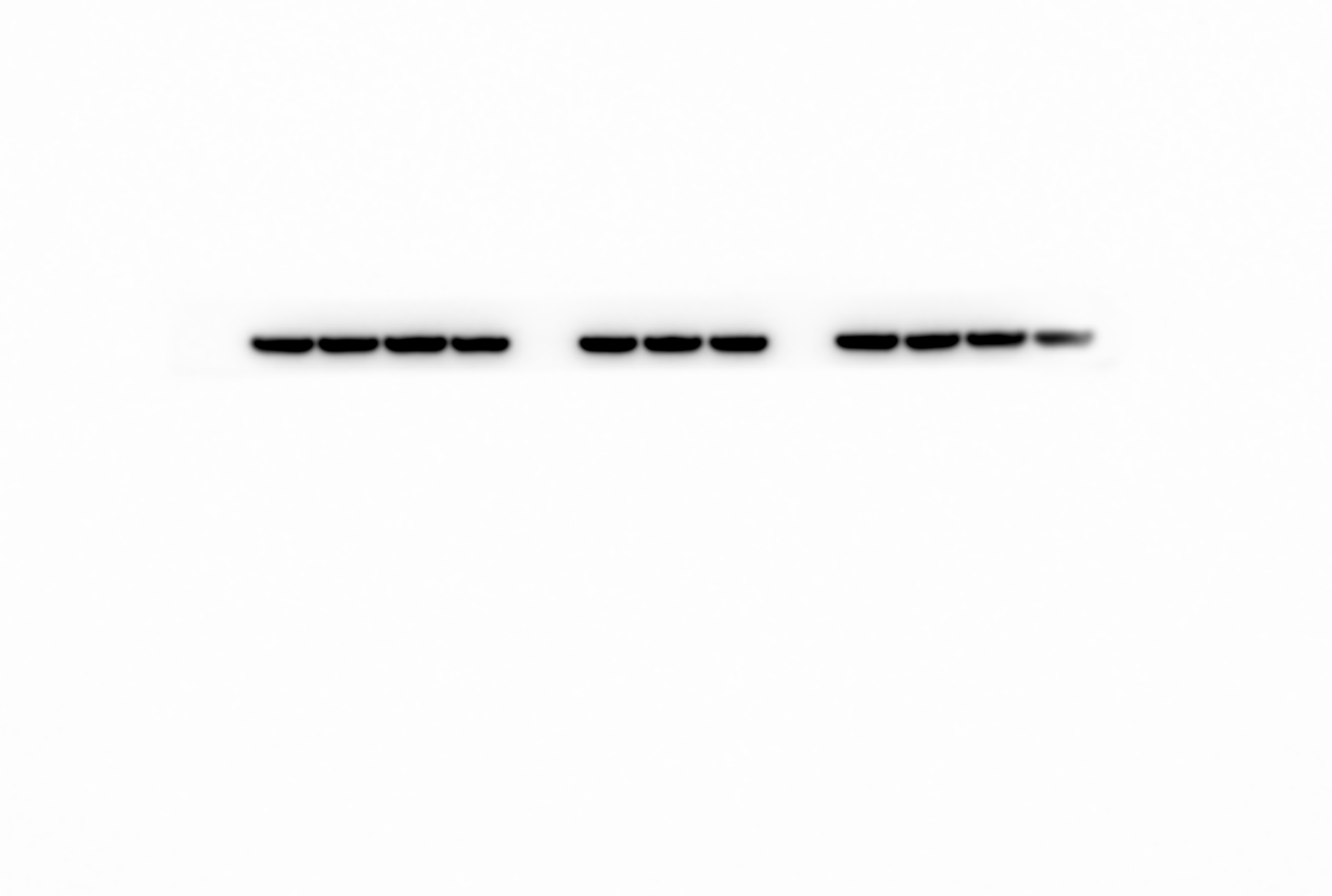

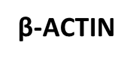

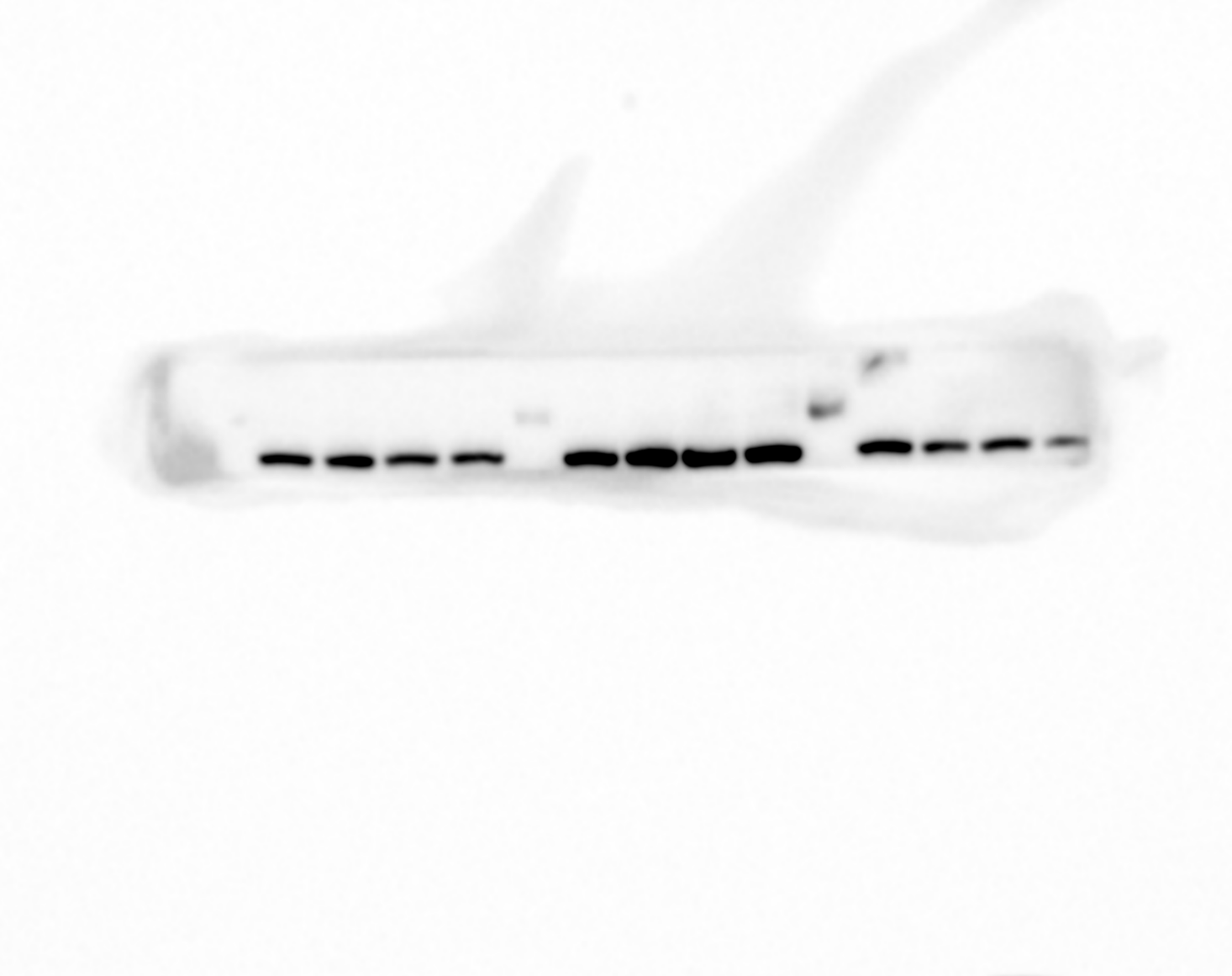

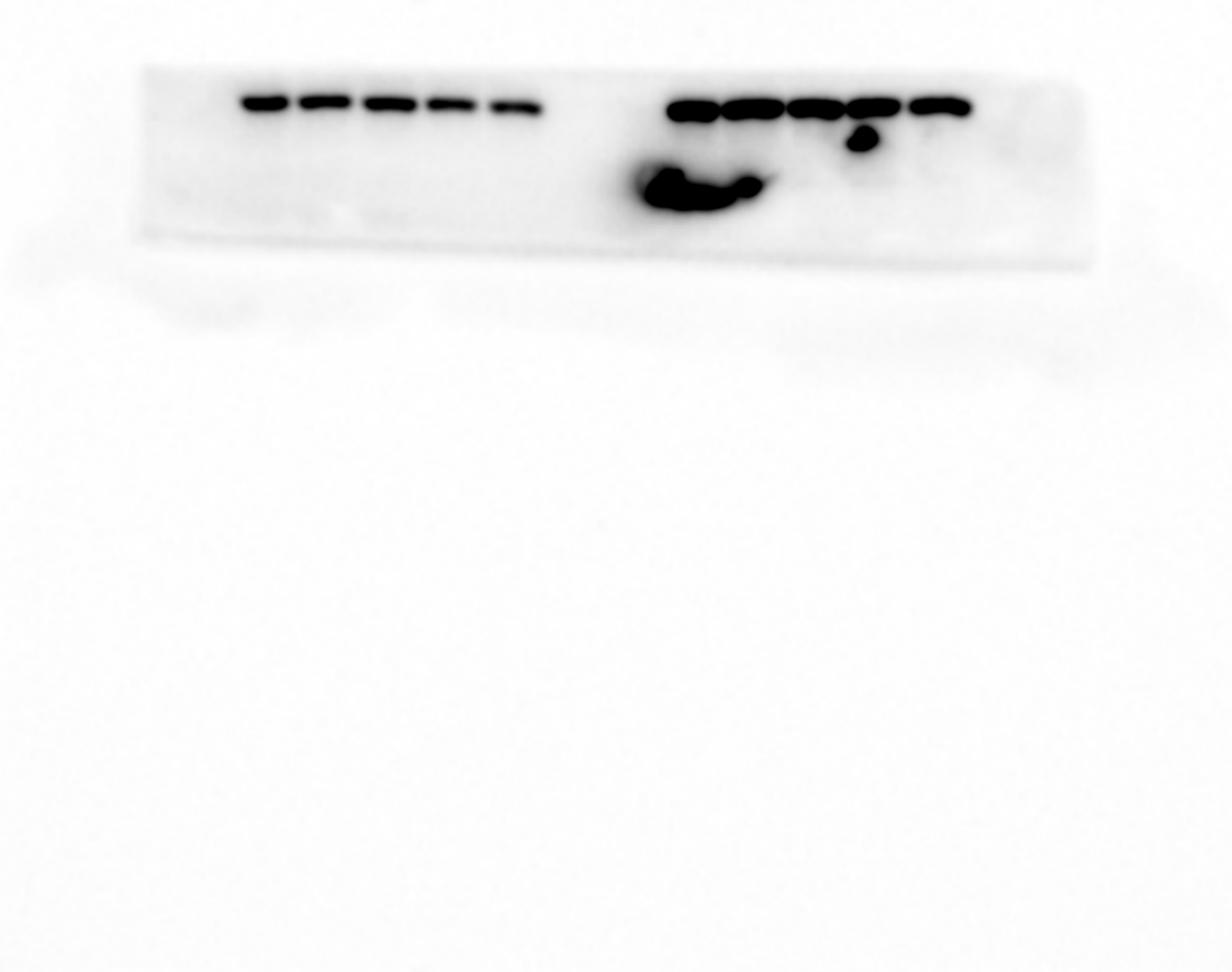

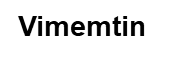

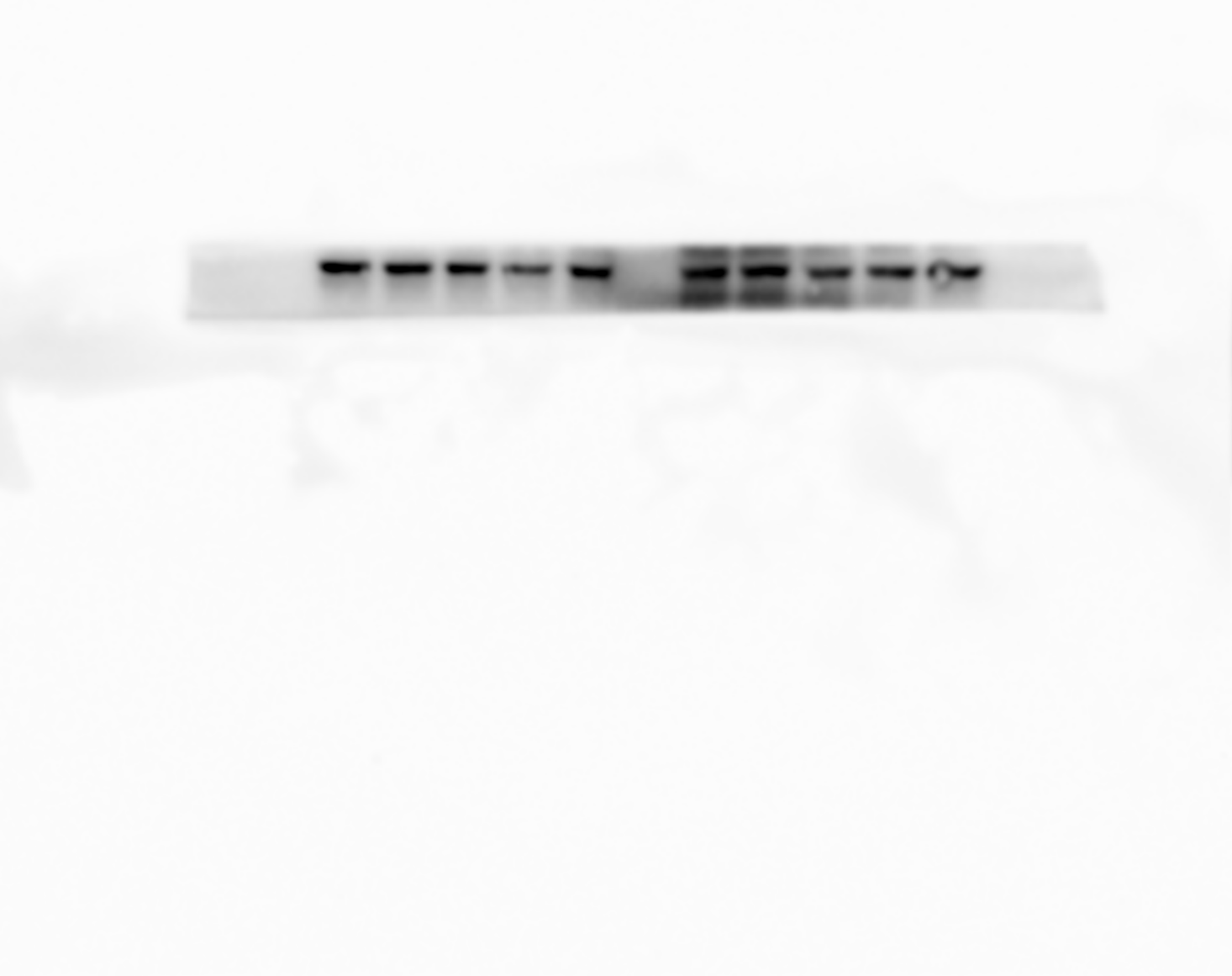

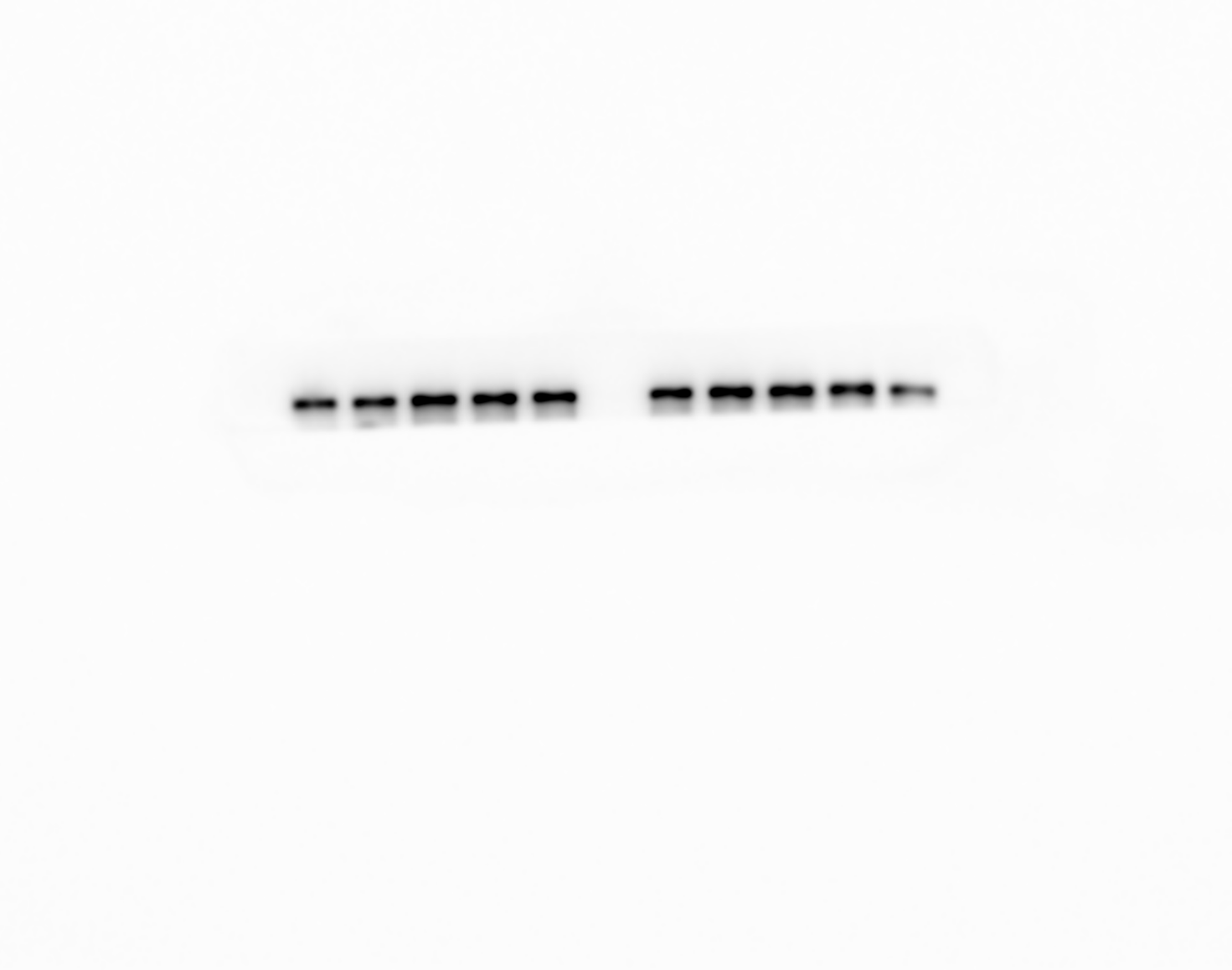

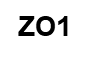

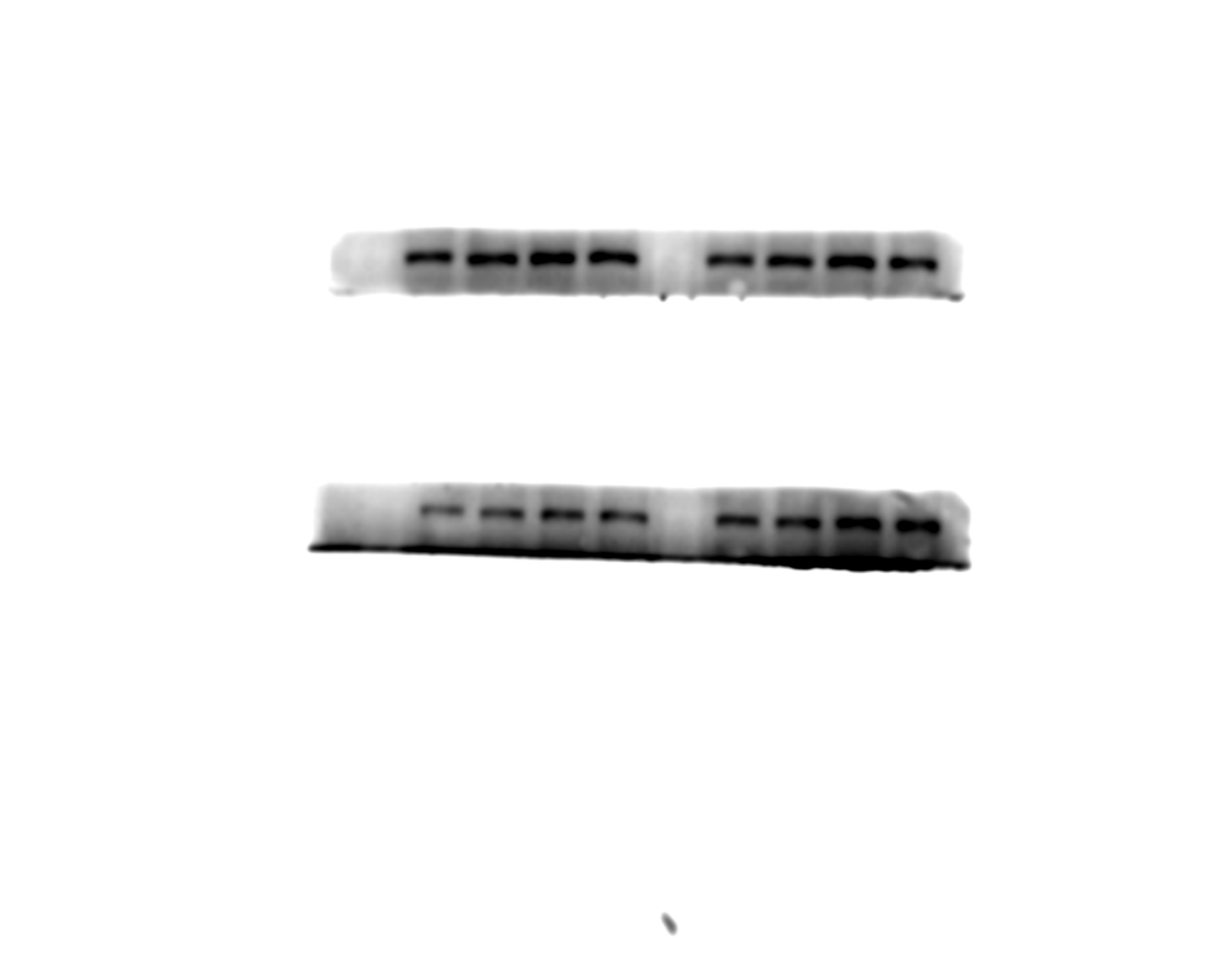

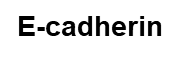

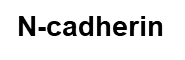


**HSC3**


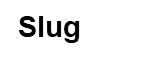

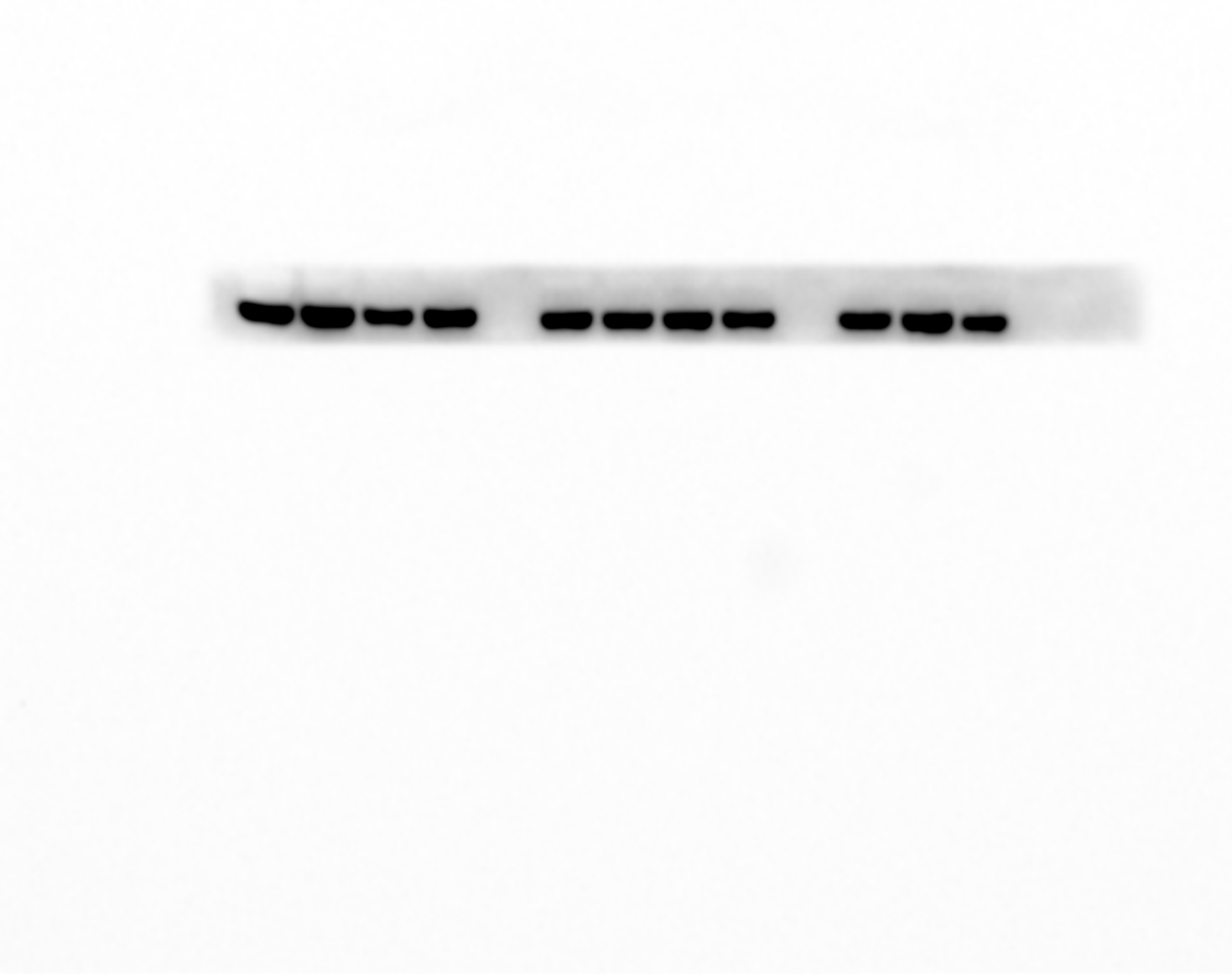


**Figure 2K**

**SCC15**


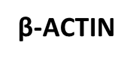

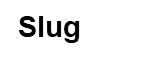

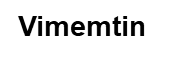

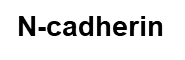

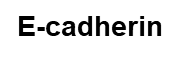

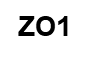

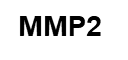

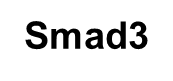

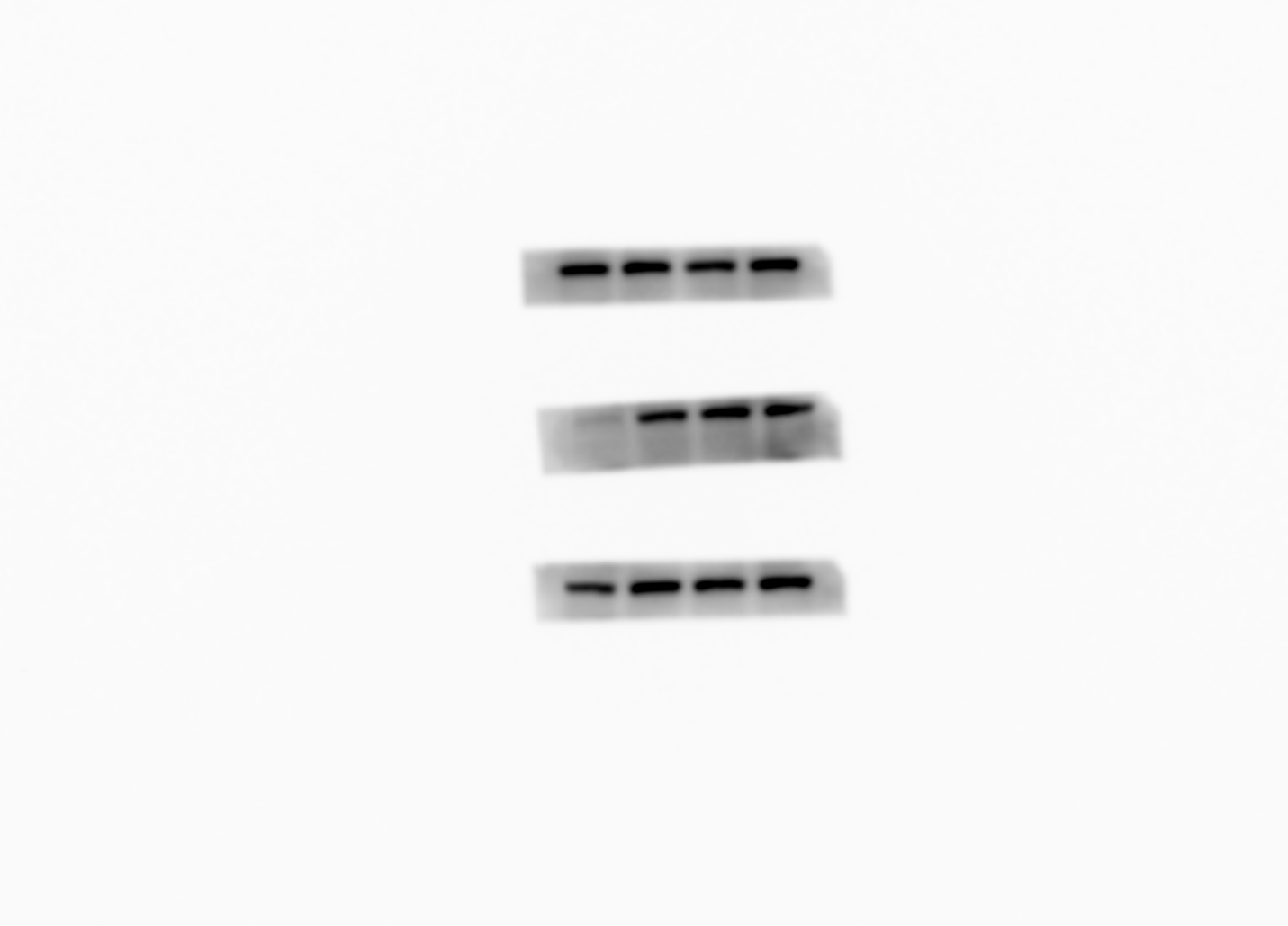

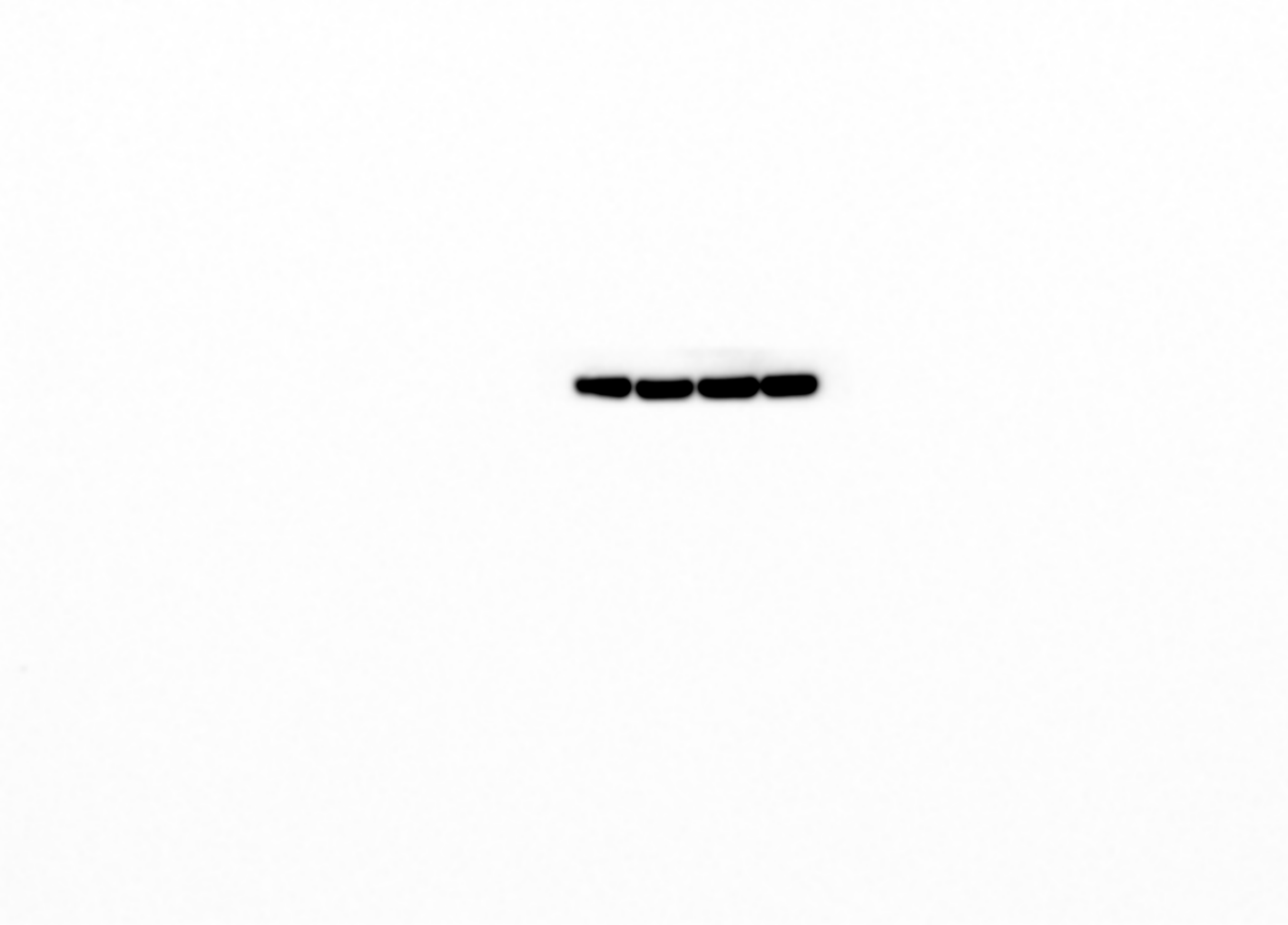

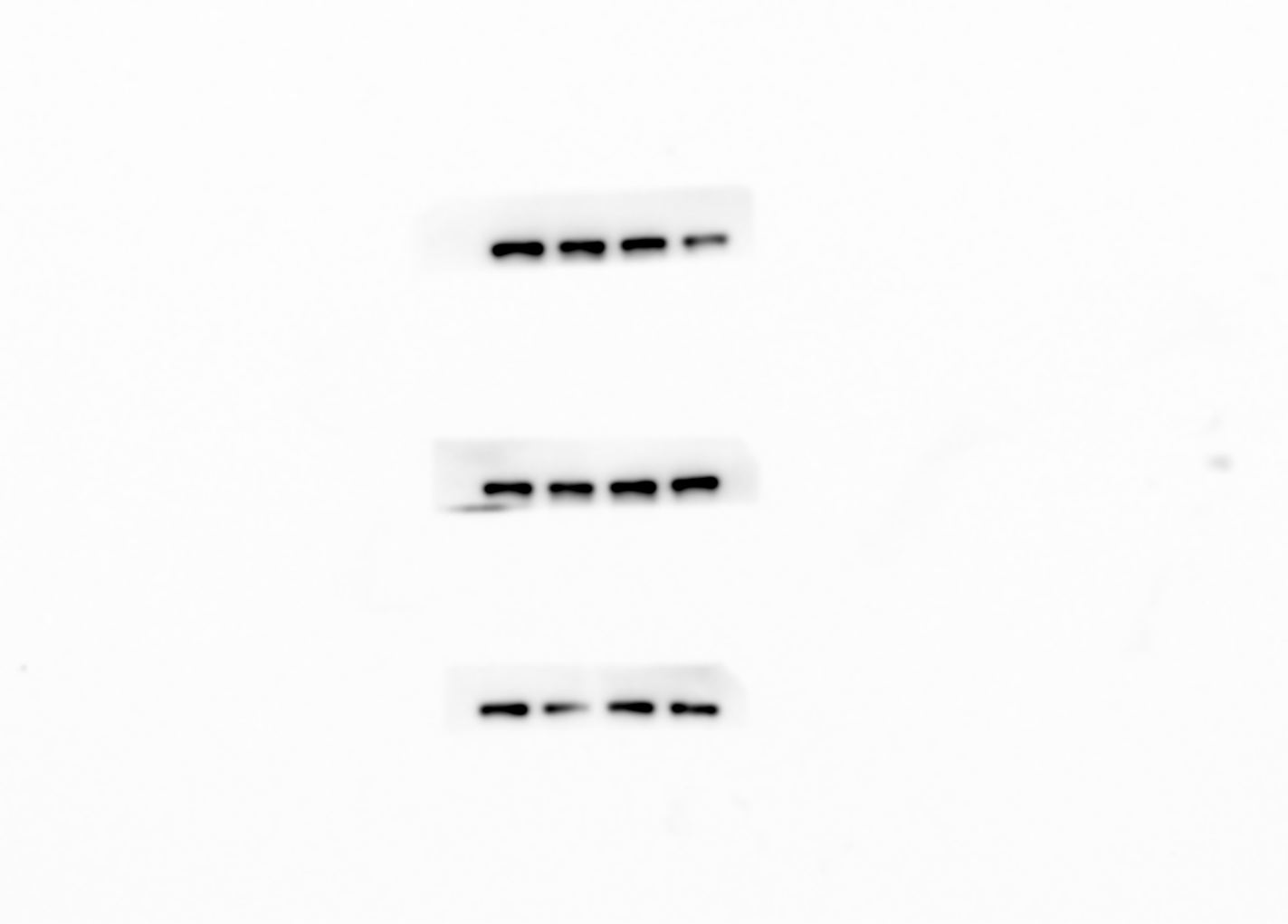

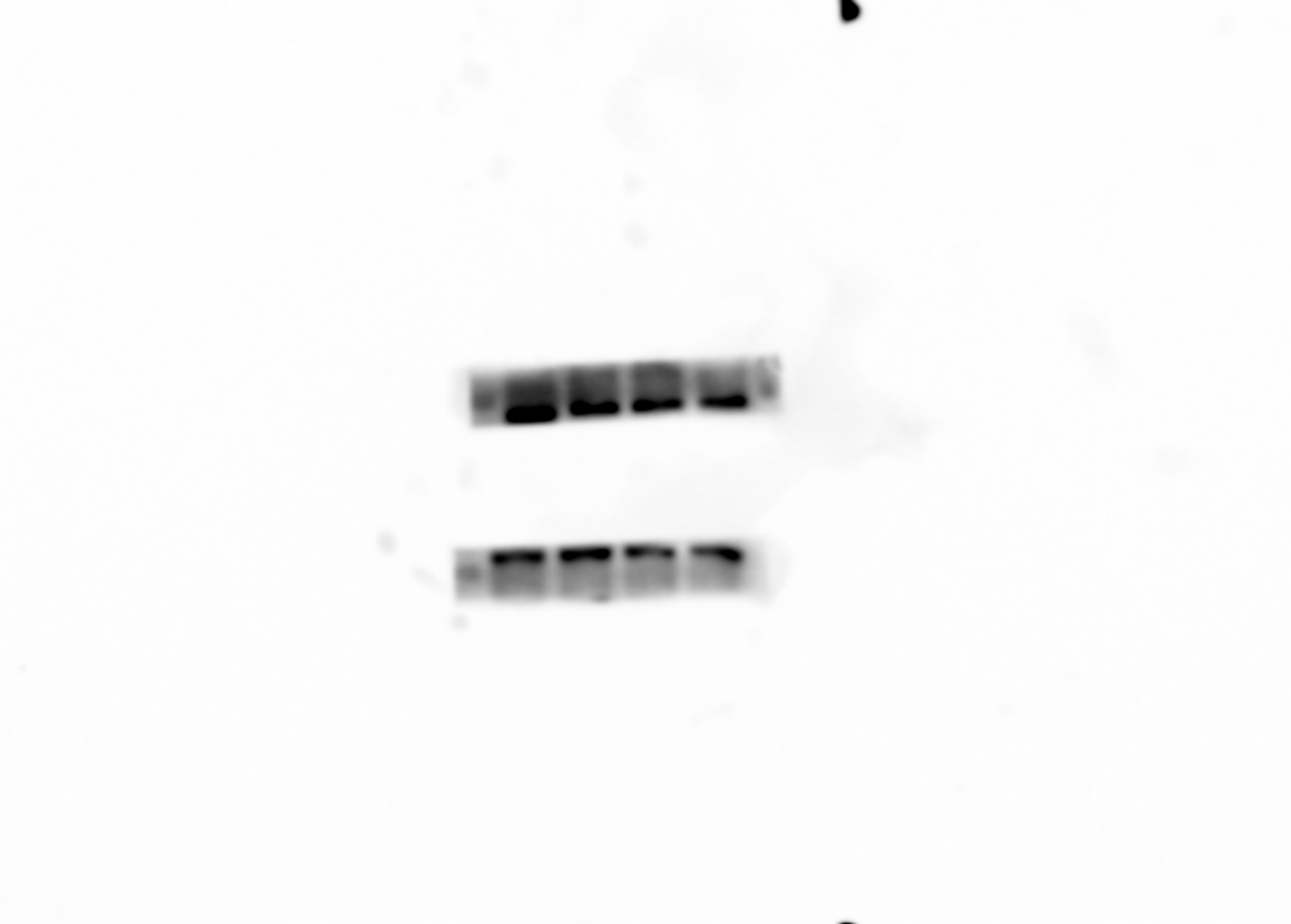

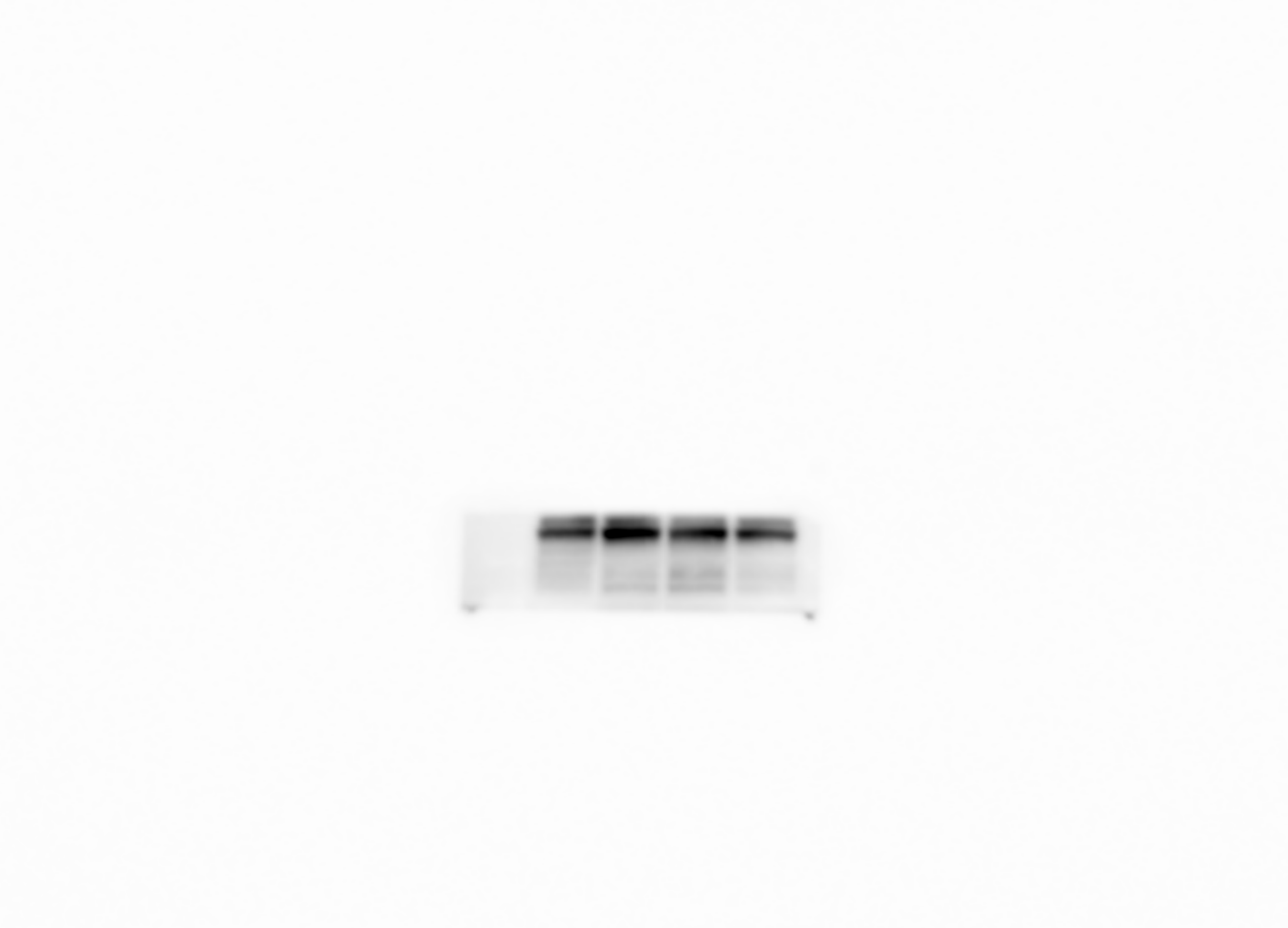

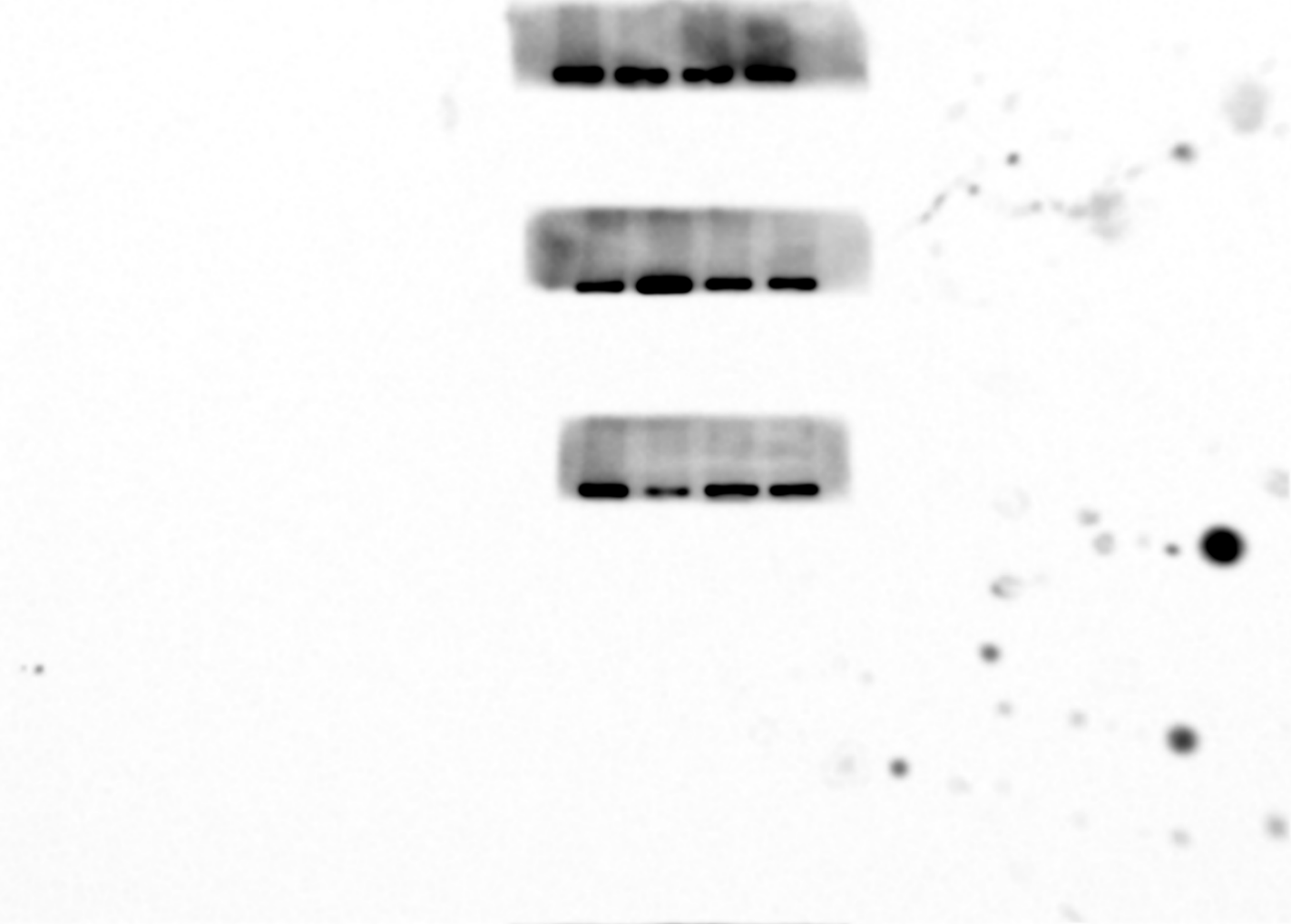

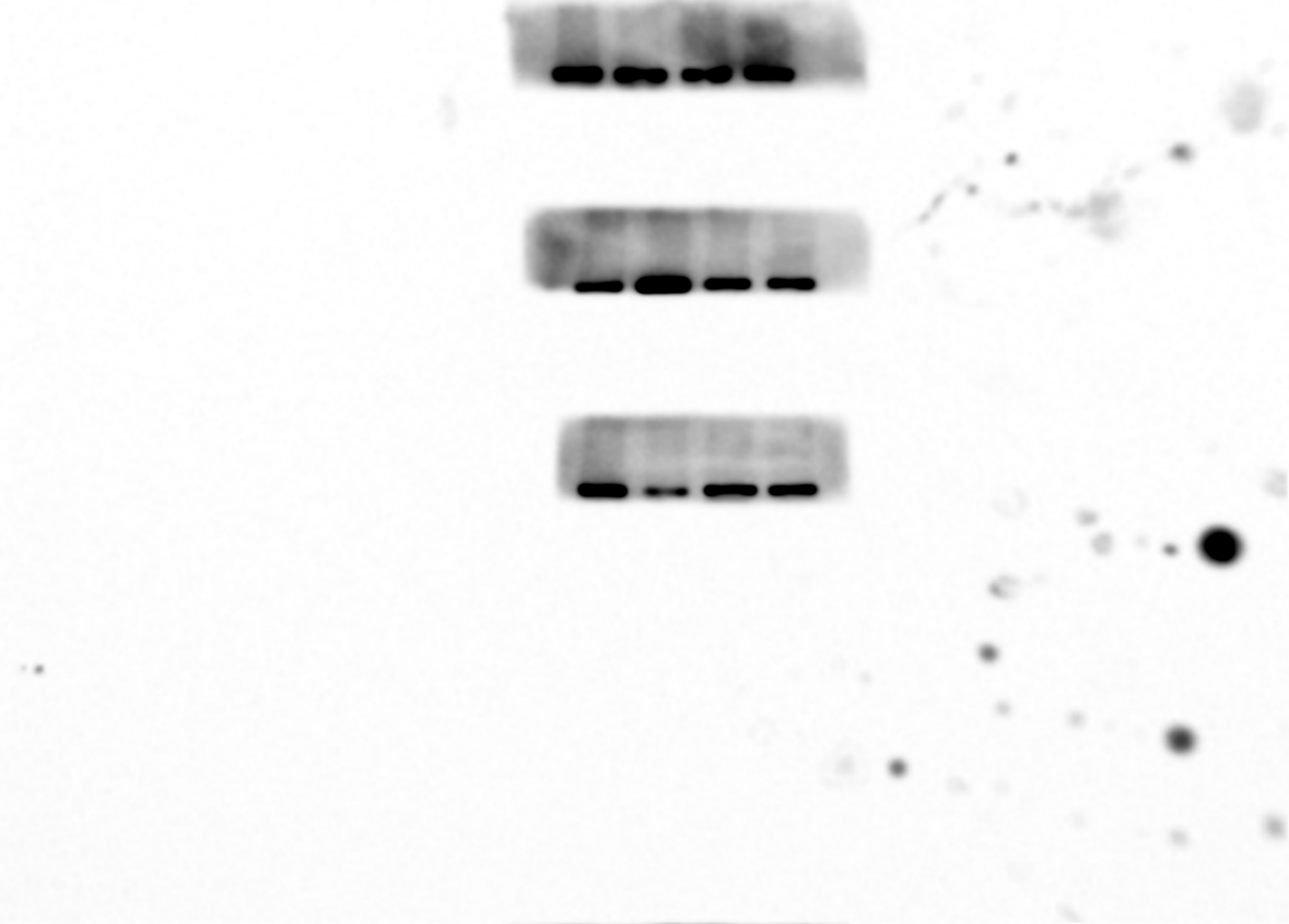

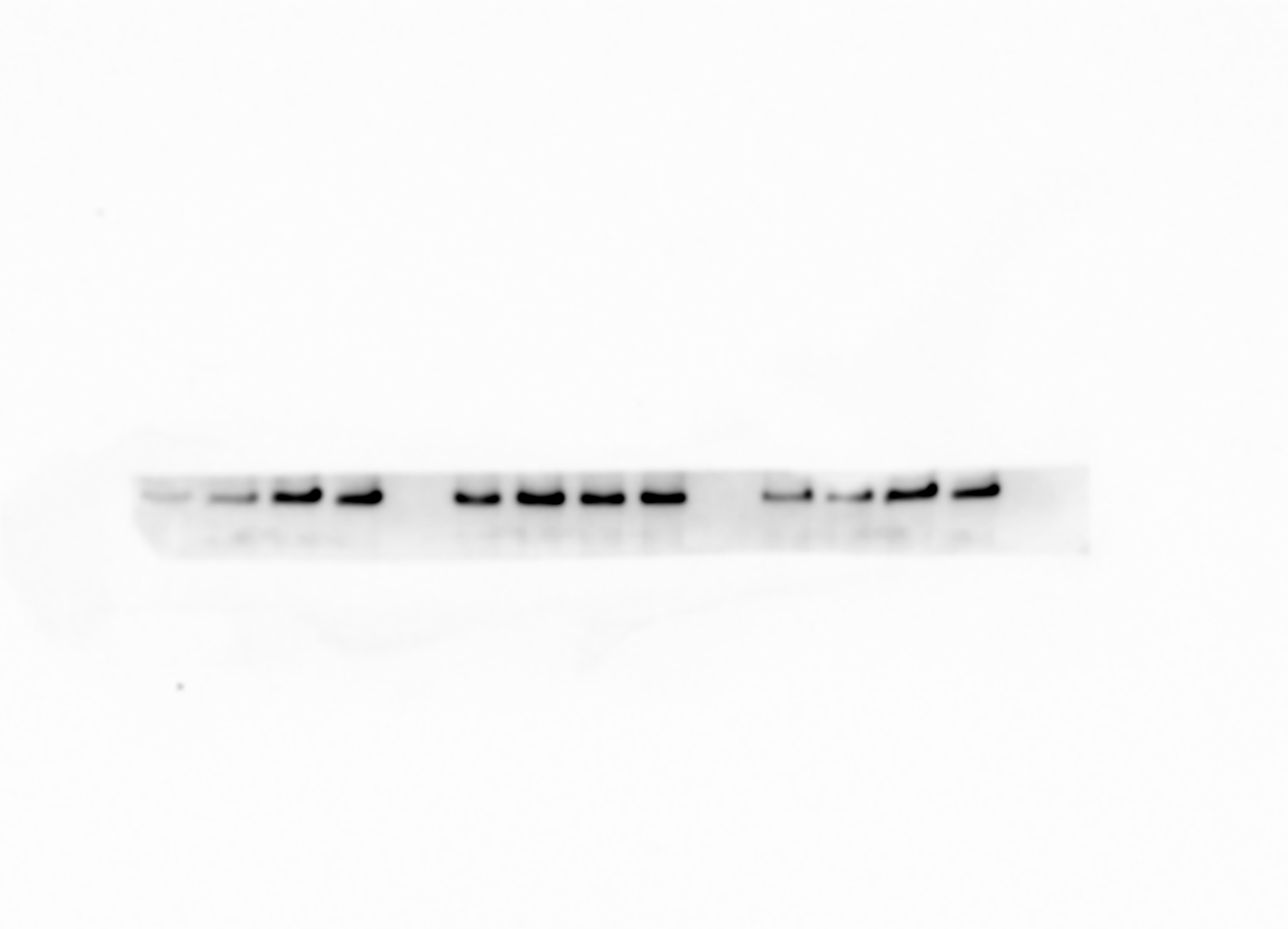


**HSC3**


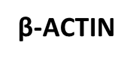

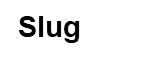

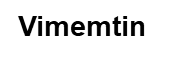

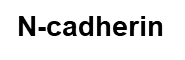

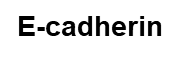

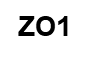

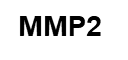

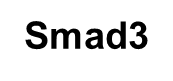

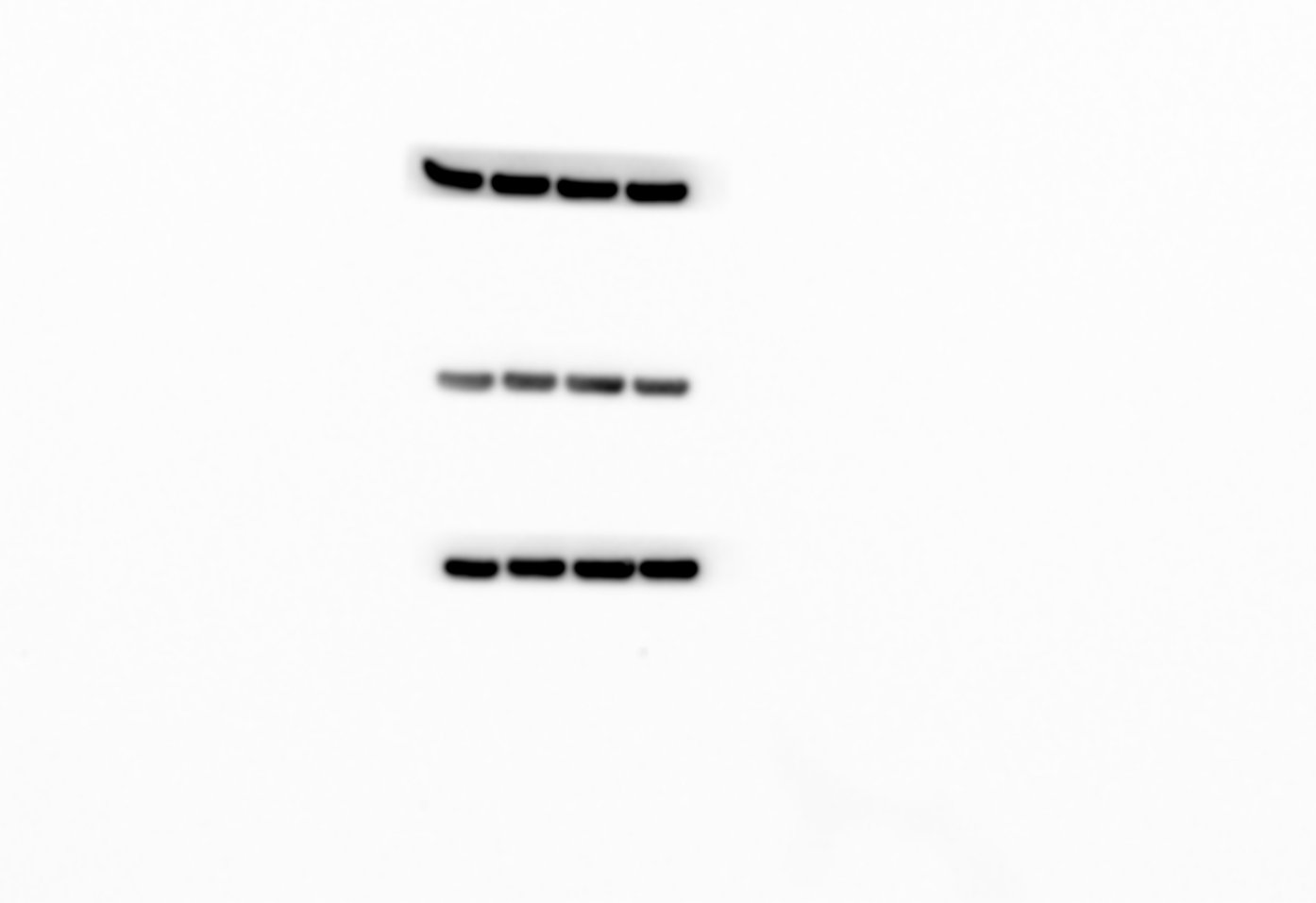

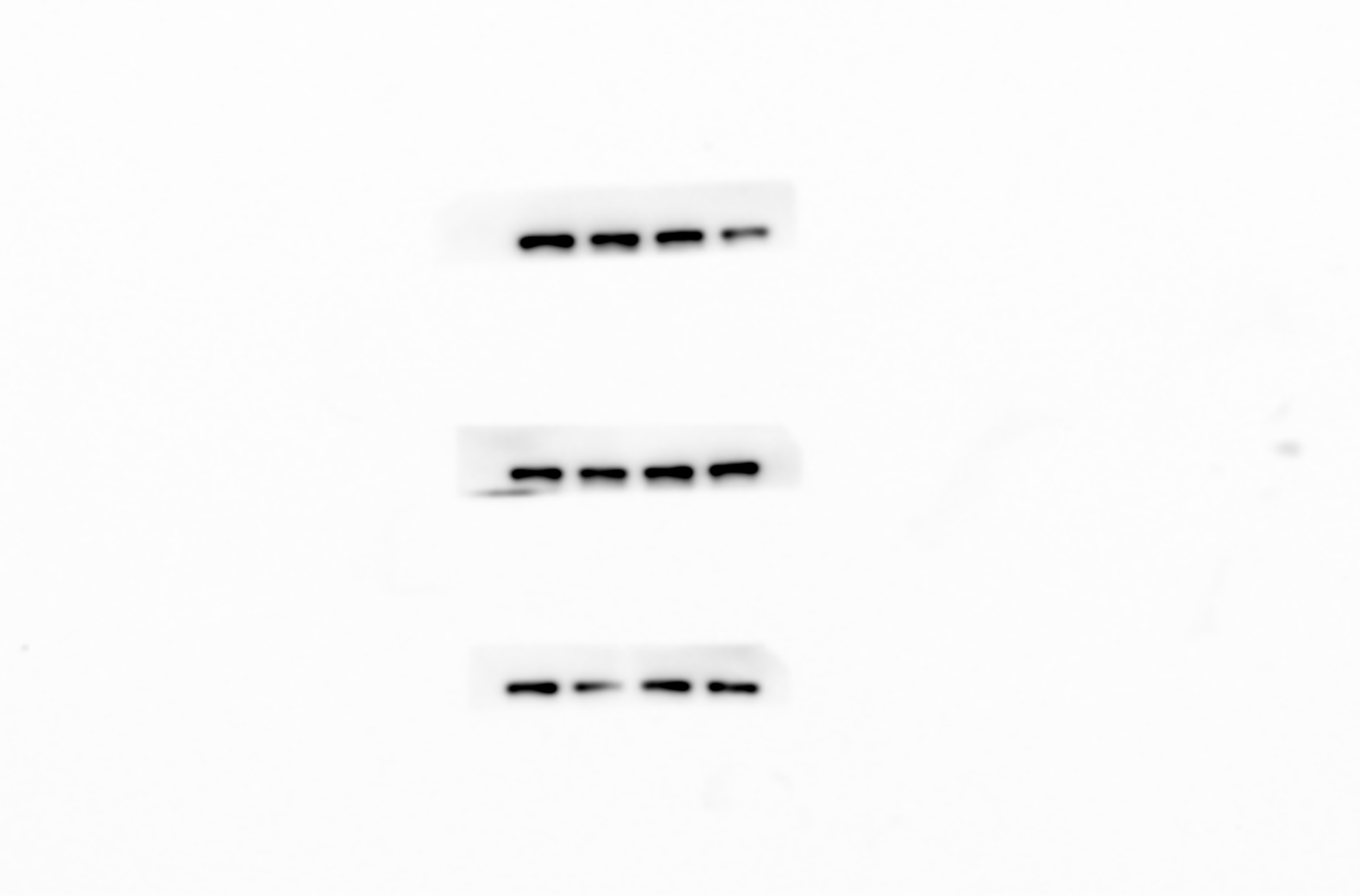

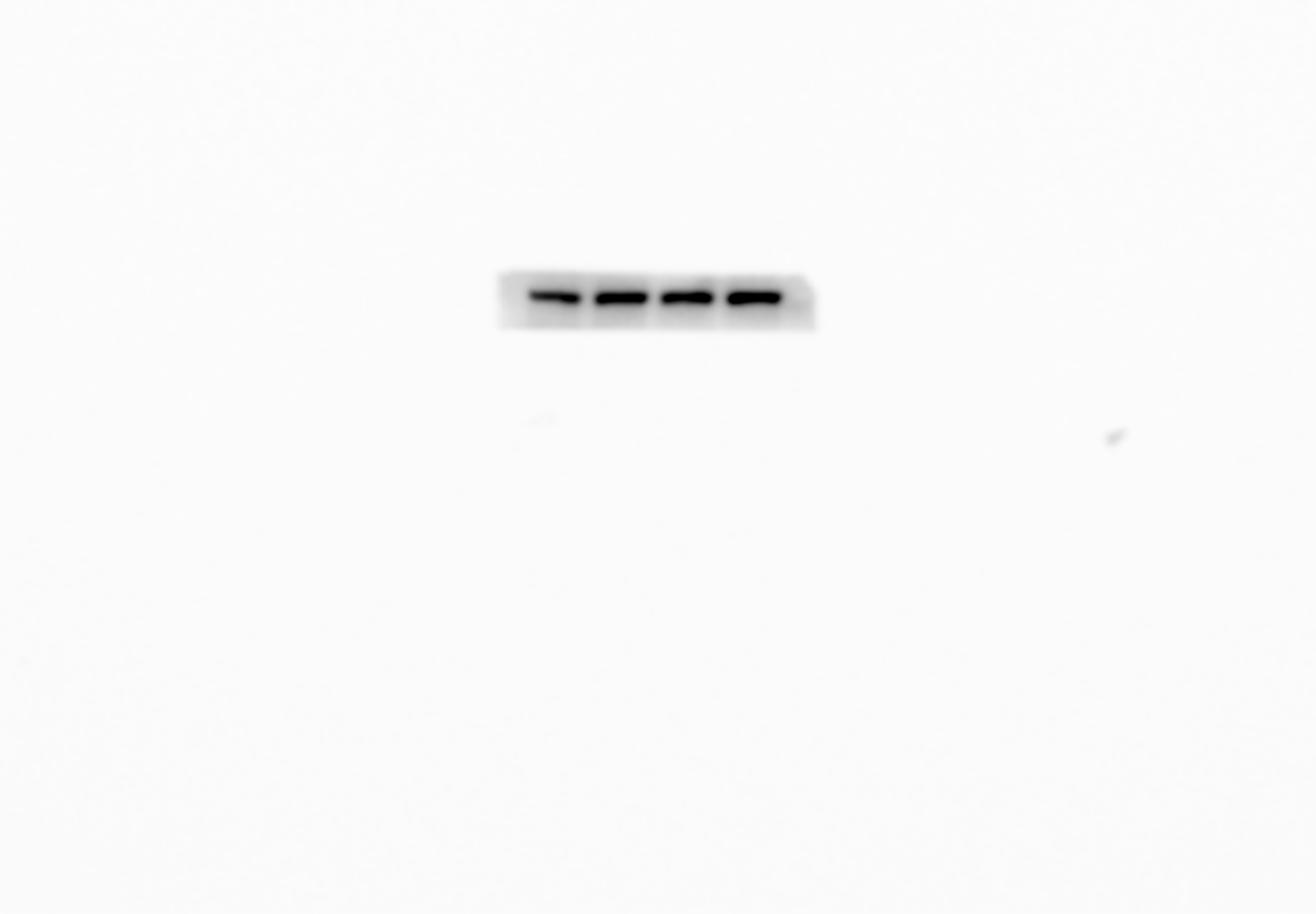

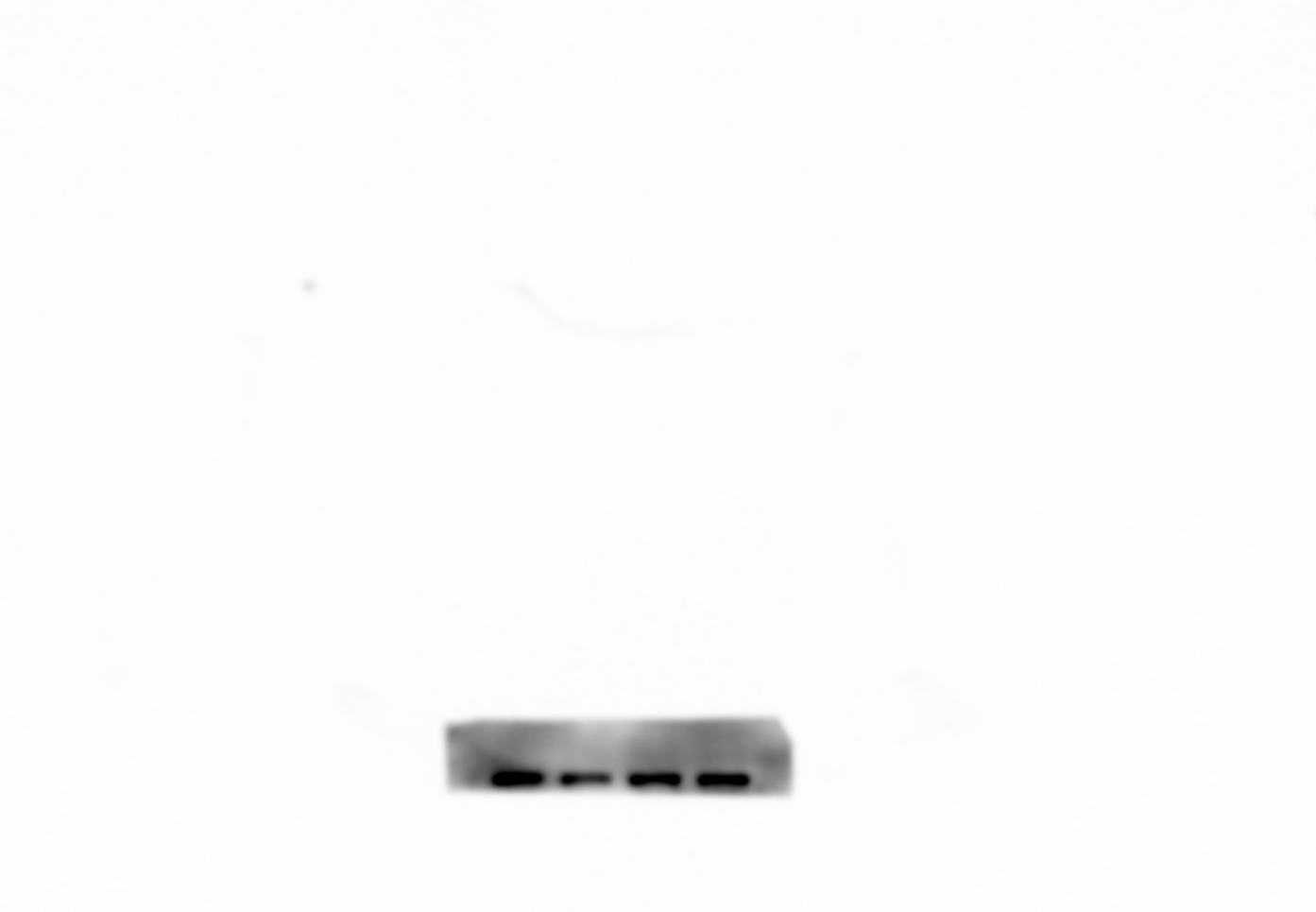

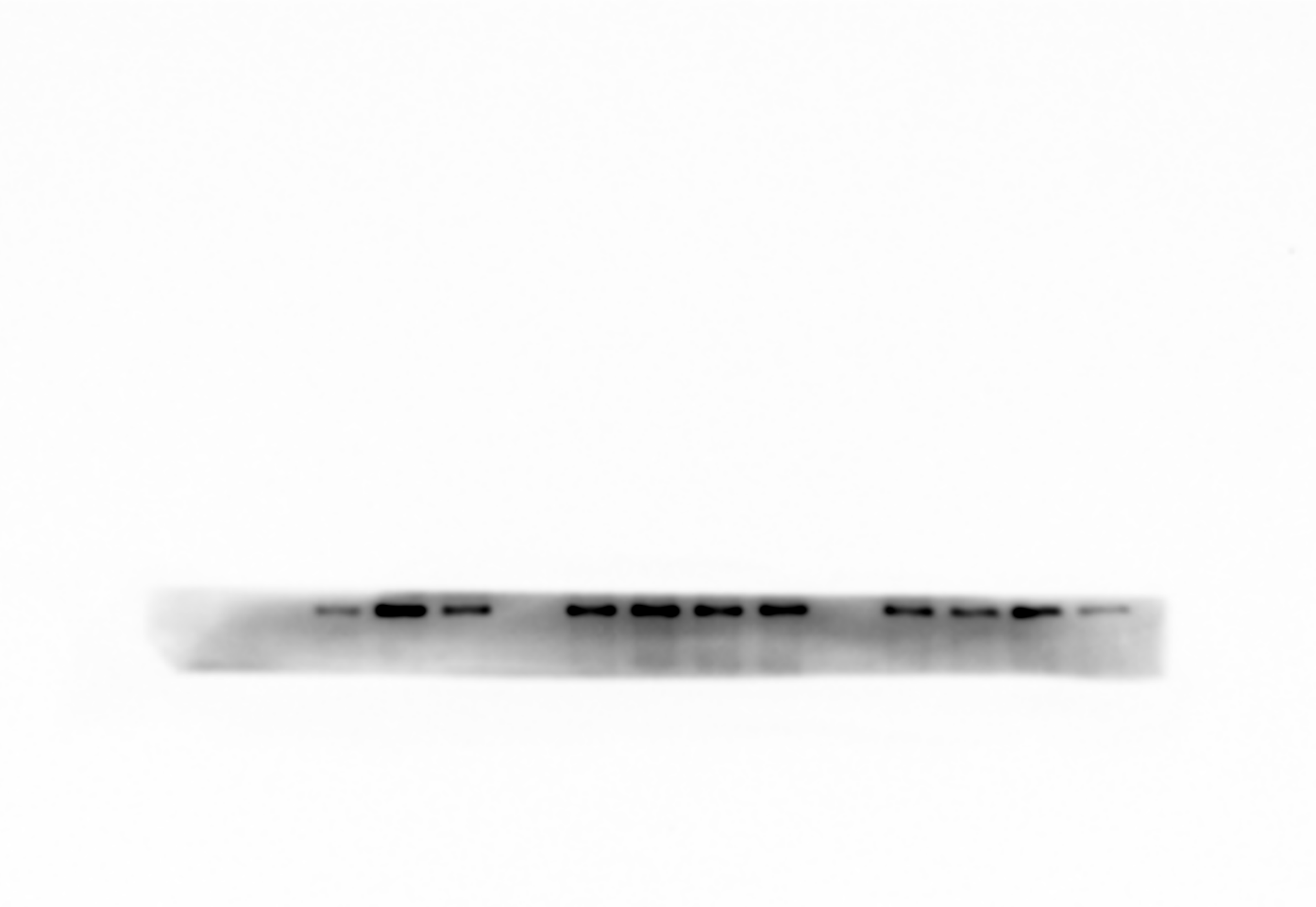

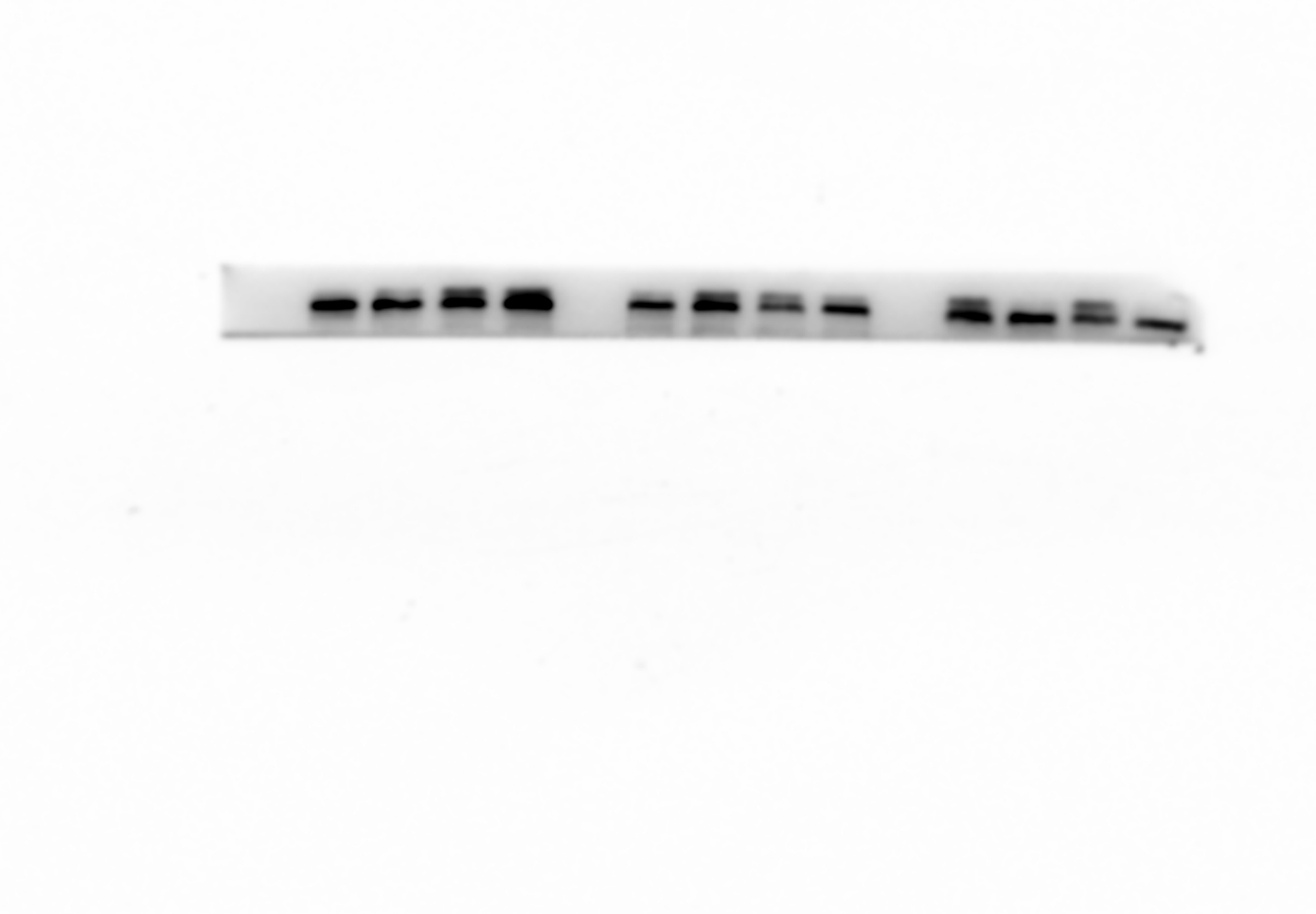

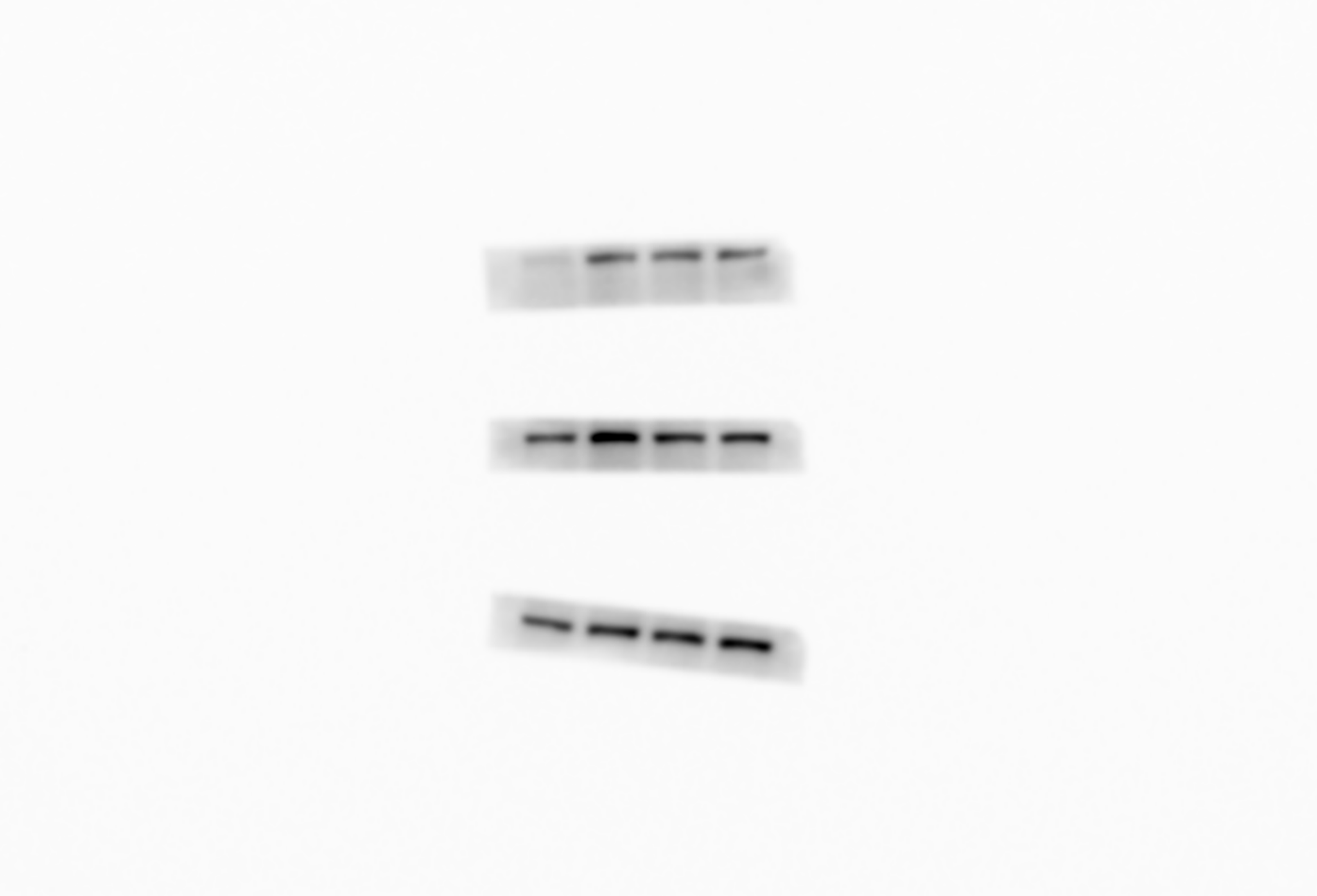

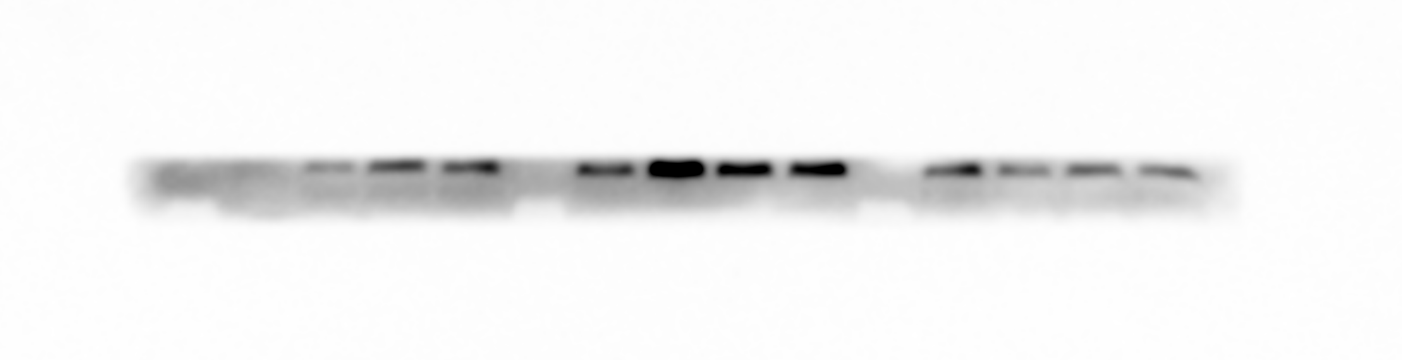


**Supplementary Figure 2.** Original images of western blot
